# Deciphering genes associated with diffuse large B-cell lymphoma with lymphomatous effusions: A mutational accumulation scoring approach

**DOI:** 10.1186/s40364-021-00330-8

**Published:** 2021-10-09

**Authors:** Sina Abdollahi, Seyedeh Zahra Dehghanian, Liang-Yi Hung, Shiang-Jie Yang, Dao-Peng Chen, L. Jeffrey Medeiros, Jung-Hsien Chiang, Kung-Chao Chang

**Affiliations:** 1 Intelligent Information Retrieval Lab, Department of Computer Science and Information Engineering, National Cheng Kung University, 701 Tainan, Taiwan; 2 Institute of Biomedical Sciences, National Sun Yat-sen University, Kaohsiung, Taiwan; 3 Department of Biotechnology and Bioindustry Sciences, College of Bioscience and Biotechnology, National Cheng Kung University, Tainan, Taiwan; 4 Department of Pharmacology, College of Medicine, National Cheng Kung University, Tainan, Taiwan; 5 University Center for Bioscience and Biotechnology, National Cheng Kung University, Tainan, Taiwan; 6 Cancer Molecular Biology and Drug Discovery, College of Medical Science and Technology, Taipei Medical University, Taipei, Taiwan; 7 Graduate Institute of Medicine, College of Medicine, Kaohsiung Medical University, Kaohsiung, Taiwan; 8 Institute of Basic Medical Sciences, College of Medicine, National Cheng Kung University, Tainan, Taiwan; 9 Kim Forest Enterprise Co., Ltd, New Taipei City, Taiwan; 10 Department of Hematopathology, The University of Texas MD Anderson Cancer Center, Houston, Texas USA; 11 Institute of Medical Informatics, National Cheng Kung University, Tainan, Taiwan; 12 Department of Pathology, College of Medicine, National Cheng Kung University Hospital, National Cheng Kung University, 138 Sheng-Li Road, 704 Tainan, Taiwan; 13 Department of Pathology, College of Medicine, Kaohsiung Medical University, Kaohsiung, Taiwan; 14 Department of Pathology, Kaohsiung Medical University Hospital, Kaohsiung, Taiwan; 15 Center for Cancer Research, Kaohsiung Medical University, Kaohsiung, Taiwan

**Keywords:** Diffuse large B-cell lymphoma, Lymphomatous effusions, Whole exome, Sequencing, Bioinformatics, HDAC1, Prognosis

## Abstract

**Introduction:**

Earlier studies have shown that lymphomatous effusions in patients with diffuse large B-cell lymphoma (DLBCL) are associated with a very poor prognosis, even worse than for non-effusion-associated patients with stage IV disease. We hypothesized that certain genetic abnormalities were associated with lymphomatous effusions, which would help to identify related pathways, oncogenic mechanisms, and therapeutic targets.

**Methods:**

We compared whole-exome sequencing on DLBCL samples involving solid organs (n = 22) and involving effusions (n = 9). We designed a mutational accumulation-based approach to score each gene and used mutation interpreters to identify candidate pathogenic genes associated with lymphomatous effusions. Moreover, we performed gene-set enrichment analysis from a microarray comparison of effusion-associated versus non-effusion-associated DLBCL cases to extract the related pathways.

**Results:**

We found that genes involved in identified pathways or with high accumulation scores in the effusion-based DLBCL cases were associated with migration/invasion. We validated expression of 8 selected genes in DLBCL cell lines and clinical samples: *MUC4, SLC35G6, TP53BP2, ARAP3, IL13RA1, PDIA4, HDAC1* and *MDM2*, and validated expression of 3 proteins (MUC4, HDAC1 and MDM2) in an independent cohort of DLBCL cases with (n = 31) and without (n = 20) lymphomatous effusions. We found that overexpression of HDAC1 and MDM2 correlated with the presence of lymphomatous effusions, and HDAC1 overexpression was associated with the poorest prognosis.

**Conclusion:**

Our findings suggest that DLBCL associated with lymphomatous effusions may be associated mechanistically with TP53-MDM2 pathway and HDAC-related chromatin remodeling mechanisms.

**Supplementary Information:**

The online version contains supplementary material available at 10.1186/s40364-021-00330-8.

## Introduction

Diffuse large B-cell lymphoma (DLBCL) is the most common form of aggressive lymphoma, accounting for approximately 40 % of all lymphoma cases in Taiwan [1], and about 33 % of cases worldwide. The outcomes of DLBCL patients are variable and therefore stratification into low-risk and high-risk groups is helpful for planning therapy. The development of body cavity effusions in DLBCL is often an indication of a significant pathologic process [2], and previous studies have shown the adverse impact of malignant lymphoma-associated effusions (direct infiltration or distant metastasis) on survival in DLBCL patients [3, 4]. Others have reported that DLBCL patients with tumorous effusions bear an even worse prognosis than patients with stage IV disease without effusions [4]. The serous fluids in body cavities circulate between parietal and visceral vessels; hence, the development of lymphoma cells in body fluids highlights the growing capability of the lymphoma cells to acquire an aggressive metastatic repertoire, including the ability to migrate, invade, and proliferate.

Conventionally, the identification of cancer behavior and cancer subtypes is attained by utilizing features derived from gene expression. With the advent of whole-genome sequencing and whole-exome sequencing, the list of biological features for identifying cancer behavior has been significantly expanded. In many studies, others have integrated whole-exome sequencing data of various DLBCL biopsy samples to distinguish genetic subtypes of DLBCL [5, 6]. In addition, several next-generation sequencing (NGS)-based studies have been published deciphering the genetic mutation profile of DLBCL subtypes [7–9]. However, the molecular mechanism underlying the emergence of lymphomatous effusions in DLBCL is largely unknown.

The accumulation of somatic mutations plays a critical role in cancer progression [10]. One of the most informative feature sets to extract the mutational signatures of different cancer subtypes is the regional mutation density (RMD) [11]. Zhang et al. calculated RMD (i.e., the number of mutations per thousand base pairs per patient) to investigate its association with different cancer subtypes. They found that genome-wide RMD profiles represent distinct patterns between melanoma and breast cancer subtypes [10]. Others have shown connections between mutation variations in megabase scale and functional genomic data obtained from tumor cell-of-origin, including chromatin accessibility and replication timing [12, 13]. Although RMD is one of the widely-used methods for detecting highlighted genes, most mutations are functionally neutral and benign. Only a small number of the mutations, which are called pathogenic, can impair molecular function and consequently lead to cancer [14]. These earlier published findings prompted us to identify the mutation profile underlying the effusion-associated lymphomas in DLBCL.

In this study, we compare the genetic profiles across two groups of patients: those with DLBCL with malignant effusions versus DLBCL patients without effusions (e.g. lymph node-based disease). For this purpose, we performed whole-exome sequencing in 9 effusion-based DLBCL patients and compared them with 22 nodal-based DLBCL samples. Our goal was to identify genes that were highly-mutated in the cases of DLBCL associated with lymphomatous effusions by investigating two factors: mutation accumulation scores and pathogenicity of mutations.

## Materials and methods

The cases of DLBCL associated with malignant effusions were derived from the National Cheng Kung University (NCKU) Hospital and included 9 cases that underwent whole exome sequencing (WES) (Table 1). All samples had a tumor load of more than 80 %. The TCGA dataset comprised WES of 22 DLBCL samples with tumors in lymph nodes (https://www.cancer.gov/about-nci/organization/ccg/research/structural-genomics/tcga). The study was approved by the institutional review board (NCKUH-A-ER-102-397 and NCKUH-A-ER-105-483) and was in accord with the Helsinki Declaration of 1975, as revised in 2013.

**Table 1:** Clinicopathologic features of DLBCL patients with lymphomatous effusions for NGS study

Case	Age	Sex	Subtype COO	Genetic	BCL2	Ki-67	c-MYC	Stage	Status
S9	56	M	ABC	EZB	+	60–70 %	+	IIIE	Alive
S10	69	M	ABC	MCD	-	60–70 %	+	IIIE	Dead
S11	71	M	ABC	EZB	+	> 90 %	+	IV	Dead
S12	52	F	GCB	EZB	-	50–60 %	-	IV	Alive
S13	45	M	GCB	N1	-	> 90 %	+	IIE	Alive
S14	39	M	GCB	BN2	+	70–80 %	-	IV	Alive
S15	63	F	GCB	UN	+	70–80 %	-	IIIE	Dead
S16	83	M	GCB	BN2	+	80–90 %	+	IVE	Dead
S17	85	F	ABC	MCD	+	> 90 %	-	IE	Dead

Abbreviations: +, positive; -, negative; Subtype of ABC (activated B-cell) or GCB (germinal center B-cell) determined by Hans cell-of-origin (COO) classification [15]. Genetic subtype based on MCD (*MYD88* and *CD79B* mutations), BN2 (BCL6 fusion and NOTCH2 mutation), N1 (NOTCH1 mutation), and EZB (EZH2 mutation and BCL2 translocation) [6]. All 9 cases had lymphomatous effusions and were negative for Epstein-Barr virus (EBV); Stage, Ann-Arbor staging system

### DNA extraction and quality control of whole exome sequencing (WES)

7Libraries were prepared according to Illumina’s instructions accompanying the DNA Sample Kit (Part# 0801 − 0303). Briefly, DNA was end-repaired using a combination of T4 DNA polymerase, E. coli DNA Pol I large fragment (Klenow polymerase) and T4 polynucleotide kinase. The blunt, phosphorylated ends were treated with Klenow fragment (32 to 52 exo minus) and dATP to yield a protruding 3- ‘A’ base for ligation of Illumina’s adapters which have a single ‘T’ base overhang at the 3’ end. After adapter ligation DNA was PCR amplified with Illumina primers for 15 cycles and library fragments of ~ 250 bp (insert plus adaptor and PCR primer sequences) were band isolated from an agarose gel. The purified DNA was captured on an Illumina flow cell for cluster generation. Libraries were sequenced on the Genome Analyzer following the manufacturer’s protocols.

### Library preparation and sequencing

For the generation of standard exome capture libraries, we used the Agilent SureSelect XT HS Reagent kit protocol for Illumina Hiseq paired-end sequencing library (catalog#G9704K). In all cases, the SureSelect XT Clinical Research Exome Version 2 (67.29Mbp) probe set was used. We used 1 ug genomic DNA to constructed library with Agilent SureSelect XT Reagent kit. The amplification adapter-ligated sample was purified using Agencourt AMPure XP beads (Beckman Coulter, Brea, CA, USA) and analyzed on a TapeStation 4200 D1000 screentape. 500 ~ 1000 ng of the gDNA library was prepared for the hybridization with the capture baits, and the sample was hybridized for Agilent hybridization program, captured with the Dynabeads MyOne Streptavidin T1 (Life Technologie, USA) and purified using a Agencourt AMPure XP beads. Use the Agilent protocol to addition of index tags by post-hybridization amplification. Finally, all samples were sequenced on Illumina NovaSeq 6000 Sequencer using 150PE protocol. The bioinformatics analyses and methods for mutation interpreters are detailed in the Supplementary Methods.

### Mutation accumulation score

Since the main purpose of this study was to compare two populations of DLBCL samples, we designed a population-based scoring method by utilizing mutation accumulation across all of the samples. For a given gene, we recorded all mutations that occurred in different samples of the population of interest. In other words, we maintained a list for each mutation that represented the samples hosting the mutation (Supplementary Figure S1A). Each list of a mutation contained *n* rows, where *n* represented the number of samples in a population. If a mutation existed in a sample, the sample’s corresponding row of the mutation list held the allele frequency of the sample. Otherwise, number *0* was assigned to the sample’s corresponding row in the list. Particularly, ${AF}_{j,i}$ represented the allele frequency of variant $i$ in sample $j$. Since we considered the mutation accumulation, we took into account the positional distances of the mutations (the number of base pairs between two mutations). Mutations that occurred in a region, bin, of a gene had a similar functional impact compared to mutations in other regions [16–18]. Previously, WinBinVec, a deep learning-based model, revealed that the mutations of a bin have similar functional impact using a one-dimensional convolutional neural network [19]. Domanska et al. [20] showed that at small-sized bins, the very high concentration of mutations made the kataegis, localized hypermutation, region stand out clearly. Accordingly, we hypothesized that if a mutation in a sample has a very short distance to another sample’s mutation, then they might have a similar functional impact. On the other hand, if the distance of the two mutations is long, then these mutations will likely not have common functional impact. Based on the hypothesis and the allele frequency values, we proposed the following equation to assign a score to each mutation:

$${Acc}_{score}^{\left(i\right)}=\frac1n\sum_{k=1}^n{AF}_{k,i}+\frac1{log\left(d_{min}\left(i\right)+1\right)+1},$$

where ${Acc}_{score}^{\left(i\right)}$ is the accumulation score of mutation $i$, $n$ is the number of samples in a population and ${d}_{min}\left(i\right)$ is the minimum distance of mutation $i$ with its vicinity mutations (mutations $i+1$ and $i-1$):

$${d}_{min}\left(i\right)=min\left[{d}_{\left(i,i-1\right)},{d}_{\left(i,i+1\right)}\text{ }\right],$$

where ${d}_{\left(i,j\right)}$ is the distance of mutations $i$ and $j$ in base pairs (bp). Finally, to obtain the accumulation score of a gene, we selected the maximum accumulation score across its mutations:

$${Acc}_{score}^{\left[g\right]}=\underset{i=1\dots m}{max}{Acc}_{score}^{\left(i\right)},$$

where $m$ is the number of mutations occurred in gene $g$. The accumulation score of a gene represented its significance in a population.

### Pathogenicity score of mutated genes

In this approach, unlike the mutation accumulation method, each sample was examined individually. A variety of mutation interpreters and databases have been designed to help understand the functional significance of genetic variants concerning their potential impact on genes and cancers. These tools can distinguish pathogenic mutations from benign ones using protein sequence, biochemical characteristics, and evolutionary information. Each mutation interpreter leveraged different methods and resources to predict the pathogenicity of a mutation. For this reason, a mutation interpreter might identify a mutation as benign, while another interpreter might detect it as a pathogenic mutation. In this study, we gathered the results of four different mutation interpreters (see Supplementary Methods). Our method prioritized pathogenic mutations rather than benign ones. For instance, in Supplementary Figure S1B, the first mutation was reported as benign (-7.8), benign (-6.2), pathogenic (6.1), and pathogenic (5) by InterVar, ClinVar, SIFT, and CADD, respectively. Next, we selected the highest score (most pathogenic) as the final mutation score (e.g., for the first mutation, 6.1 is the highest score). Finally, for calculating a gene score, we summed up all mutation scores in which their pathogenicity scores were higher than 0 (pathogenic). The use of a consensus method for assigning pathogenicity score to each gene was modified from the CoLaSp model [21].

### Gene microarray analysis

The study cohort was composed of two cases of DLBCL in effusions (40 y/o male with GCB-type DLBCL in ascites and 55 y/o male with ABC-type DLBCL in pleural effusion) and two cases of DLBCL in solid organs (34 y/o female with ABC-type DLBCL in anterior mediastinum and 36 y/o female with GCB-type DLBCL in mediastinal lymph node). Total RNA was extracted by Trizol Reagent (Invitrogen, USA) according to the instruction manual. Purified RNA was quantified at OD260 nm by using a ND-1000 spectrophotometer (Nanodrop Technology, USA) and qualified by using a Bioanalyzer 2100 (Agilent Technology, USA) with RNA 6000 nano labchip kit (Agilent Technologies, USA). Agilent microarray hybridization chamber kits were used for experiments (G2534A, Agilent Technologies, Palo Alto, CA, USA). Total RNA from DLBCL cells was used to prepare biotinylated RNA according to the manufacturer’s recommendation. Ratios for GAPDH and β-actin (3’/5’) were within acceptable limits. After RNA isolation, two aliquots of 0.2 µg of RNA were linearly amplified and fluorescently labeled with either Cy3-CTP (DLBCL in effusions) or Cy5-CTP (DLBCL in lymph nodes) with the Agilent Low Input Quick Amp Labeling Kit (Agilent Technologies). Equal amounts (0.3 µg) of cyanine-labeled samples were hybridized to Agilent 8 × 60 K Microarray chip (Agilent Technologies) according to the manufacturer. The microarray was scanned using an Agilent Microarray Scanner, and the scan was quantified using Agilent Feature Extraction software (version 10.5.1.1) and normalized using Rank consistency linear LOWESS with minimum background correction. Differentially expressed gene sets were identified using significance analysis of microarrays (SAM) and only those with positive or negative changes of 2.0-fold or more were included. Hierarchic clustering was performed.

### Bioinformatics and pathway analysis

Gene Set Enrichment Analysis (GSEA, UC San Diego, CA, USA) was used to determine the enriched genes from the cDNA microarray of DLBCL in effusions vs. DLBCL in solid organs. An enriched score was generated for each priori defined gene set based on the number of enriched genes. The settings and parameters are described in Table 2. According to an enriched score, we identified the gene sets that were positively and negatively correlated with the input data. Ingenuity Pathway Analysis software (IPA, Qiagen, Redwood City, CA, USA) was used to perform pathway analysis by subjecting selected genes. Known functional networks were tested for enrichment based on canonical pathways, relationship to upstream regulators, molecular and cellular functional groups, and associated network functions.

**Table 2:** The parameters and the settings in Gene Set Enrich Analysis (GSEA)

Chip Platform	Human_Gene_Symbol_with_Remapping_MSigDB.v7.4.chip
Gene sets database	C2.cp.kegg.v7.4.symbols.gmt [Curated]
Number of permutations	1000
Permutation type	gene_set
Enrichment statistics	Weighted
Metric for ranking genes	Signal2Noise
Min size (exclude smaller sets)	15
Max size (exclude larger sets)	500
Collapsing mode for probe sets $\ge 1$ gene	Max_probe
Normalize mode	meandiv

### DLBCL cell lines and EBV-transformed lymphoblastoid cell lines (LCL)

To validate the clinical significance of genes yielded from the above bioinformatic analyses, quantitative real-time PCR, Western blotting, and immunohistochemical analysis on clinical samples were performed. The DLBCL cell lines and LCL (Supplementary Table S1) were cultured at 37 °C and 7 % CO_2_ in RPMI 1640 medium (Gibco/BRL, Grand Island, NY, USA) supplemented with 10 % heat-inactivated fetal bovine serum (FBS), 4 mM of glutamine, 75 units/ml of streptomycin, and 100 units/ml of penicillin. Cell viability was determined using the trypan blue exclusion test or MTT (3-[4,5-dimethylthiazol-2-yl]-2,5-diphenyltetrazolium bromide) assay.

### TaqMan quantitative real time PCR (TaqMan qRCR)

Total RNA of *B*-*cell* lymphomas and cells from pleural fluid of lymphoma patients were extracted by QIAzol (#79,306, QIAGEN) according to the manufacturer’s instruction. One microgram of total RNA was reverse transcribed into cDNA using High-Capacity cDNA Reverse Transcription Kit (#4,368,814, Thermo). Gene expression was determined by TaqMan quantitative real-time polymerase chain reaction (qPCR) using ChamGE Probe qPCR Master Mix (CGE-03, TopGen Biotech). Sequences of PCR primers and TaqMan probes are listed as follows:

**Table:** 

TP53BP2	Probe	ACAAACTTGCGTAAAACTGGCTC
	Forward	AAGACTCGGTGAGCATGCG
	Reverse	CCTCATTCCATGAGCGATACG
SLC35G6	Probe	AGGAAAGATGGCTGGCAGTC
	Forward	CACTCCAACCATGTCACAATGG
	Reverse	GTCAGGCGGGTTCAAGTAGG
PDIA4	Probe	ACTGAAGCCAGTCATCAAATCCC
	Forward	CATGGAGCCAGAGGAGTTTGAC
	Reverse	GACGGGTCCCTTGTTGTTCTT
MUC4	Probe	AGCTCTTTGAGAATGGGACGTTG
	Forward	CAATGCTGAGGATGCCAACTT
	Reverse	TGCTAGAATCTCCAGAGTGAATGG
MDM2	Probe	AGAATTGGCTTCCTGAAGATAAAGGG
	Forward	CACTTCATGCAATGAAATGAATCC
	Reverse	TGAGTTTTCCAGTTTGGCTTTCT
IL13RA1	Probe	ACTTCCCGTGTGAAACCTGATC
	Forward	AATAATGGTCAAGGATAATGCAGGA
	Reverse	CATCATTGTGGAAGGAGAGGTTT
HDAC1	Probe	ATGGAAATCTATCGCCCTCACAA
	Forward	CTCACCGAATCCGCATGAC
	Reverse	GCTGTGGTACTTGGTCATCTCCT
ARAP3	Probe	ACTTACAGCGGCTTCCTGTACT
	Forward	CTGGCCTCTTGCCCTCAGA
	Reverse	GAGGGTCCAGCTTTGTTGCT
ACTB	Probe	AGGCACCAGGGCGTG
	Forward	ATGTGCAAGGCCGGCTT
	Reverse	CTCTTGCTCTGGGCCTCGT

### Western blot analysis

Total lysates of B*-*cell lymphomas and cells from pleural fluid of lymphoma patients were extracted by RIPA lysis buffer (50 mM Tris-HCl/pH 8.0, 150 mM NaCl, 0.5 % sodium deoxycholate, 1 % Nonidet P-40, 0.1 % SDS, 1 mM DTT, 10 mM β-glycerol phosphate, and 1 mM EGTA) supplemented with protease inhibitor cocktail (P8340, Sigma). Protein concentration is measured using Protein Assay Kit (#5,000,006, Bio-Rad). Anti-ERp72 (PDIA4) antibody (#2798, Cell Signaling), anti-TP53BP2 antibody (ab181377, Abcam) and anti-α-tubulin (T6199, Sigma,) were used for western blot analysis.

### Immunohistochemical analysis

Immunohistochemical staining was performed on deparaffinized tissue sections of formalin-fixed material, pre-treated with the Epitope Retrieval Solution 2 (EDTA, pH 9.0). The procedures were performed using the Bond-Max Automated IHC stainer (Leica Biosystems Newcastle Ltd, Australia). The primary antibodies and working dilutions were as follows: MUC4 (1:100, 8G7, mouse monoclonal, Zeta Corporation, Taichung, Taiwan), MDM2 (1:100, IF2, mouse monoclonal, Invitrogen, Thermo Fisher Scientific, Waltham, MA USA), and HDAC1 (1:400, 4E1, mouse monoclonal, GeneTex, Hsinchu, Taiwan). Appropriate positive and negative controls were used. Counterstaining was carried out with hematoxylin, and images were photographed using a digital microscope camera (DP12; Olympus Co., Tokyo, Japan) and processed by Adobe Photoshop version 8.0 software (Adobe Systems Incorporated, San Jose, CA, USA). The staining was deemed positive when tumor cells showed nuclear expression of MDM2 in ≥ 10 % [22] and HDAC1 in ≥ 50 % [23] of the tumor cells, as described previously. For MUC4, cytoplasmic staining in ≥ 10 % of the tumor cells was graded as positive. The independent cohort of additional 51 DLBCL cases with (*n* = 31) or without (n = 20) lymphomatous effusions are listed in Supplementary Table S2 [4].

### Statistical analysis

Appropriate statistical tests were used to examine the relationships and correlations between variables, including χ^2^-test. Overall survival was measured from initial diagnosis to death from any cause, with follow-up data of surviving patients assessed at the last contact date. Estimates of overall survival distribution were calculated using the method of Kaplan and Meier. Time-to-event distribution was compared using the log-rank test. The analyses were carried out using SPSS statistical software (SPSS, Inc., Chicago, IL, USA).

## Results

WES information of 9 effusion-associated DLBCL samples is provided in Supplementary Table S3. The mean depth of high-quality sequences on targets ranged from 197.4x to 377.2x, with median 301.9x. Because matched germline DNA was unavailable, somatic variants were identified by comparing with genetic variation databases. Variants present in public and in-house databases (gnomAD East Asian and Taiwan biobank) with a minor allele frequency (MAF) > 1 % were filtered out. Supplementary Figure S2 shows the number of these potentially somatic variants in each sample. A total of 3,919 single nucleotide variants (SNVs, median per sample: 476, range: 403–555) and 209 insertions/deletions (indels, median per sample: 30, range: 18–37) showed potentially protein-changing features (Supplementary Table S4 Excel file). WES data are available in the National Center for Biotechnology Information (https://submit.ncbi.nlm.nih.gov/), BioProject: PRJNA740363 (https://dataview.ncbi.nlm.nih.gov/object/PRJNA740363?reviewer=49scseu631s44tsbpgt64h0pmi).

### Mutation scoring method distinguishes higher genetic alterations in migration/invasion-associated pathways in effusion-associated DLBCL

The routes of lymphoma cells into body cavities might spread via blood or lymphatic vessels [24]. The serous fluids in body cavities circulate from parietal vessels to visceral vessels. Hence, the existence of lymphoma cells in body fluids highlights the increased capability of the lymphoma cells to spread. Furthermore, this capability might be a manifestation of a more aggressive metastatic dissemination repertoire, such as migration, invasion, and adhesion. For this reason, we extracted migration/invasion regulator genes in various pathways. The extracted genes belonged to the one of the following pathways or groups: B cell receptor (BCR), NFκB, toll-like receptors (TLR), focal adhesion kinase (FAK), BCL10-CARD11-MALT1 (BCM) complex, cytokines, somatic hypermutation, leukocyte transendothelial migration, glycoproteins, and transmembrane proteins (Supplementary Table S5 Excel file).

**Fig. 1:**
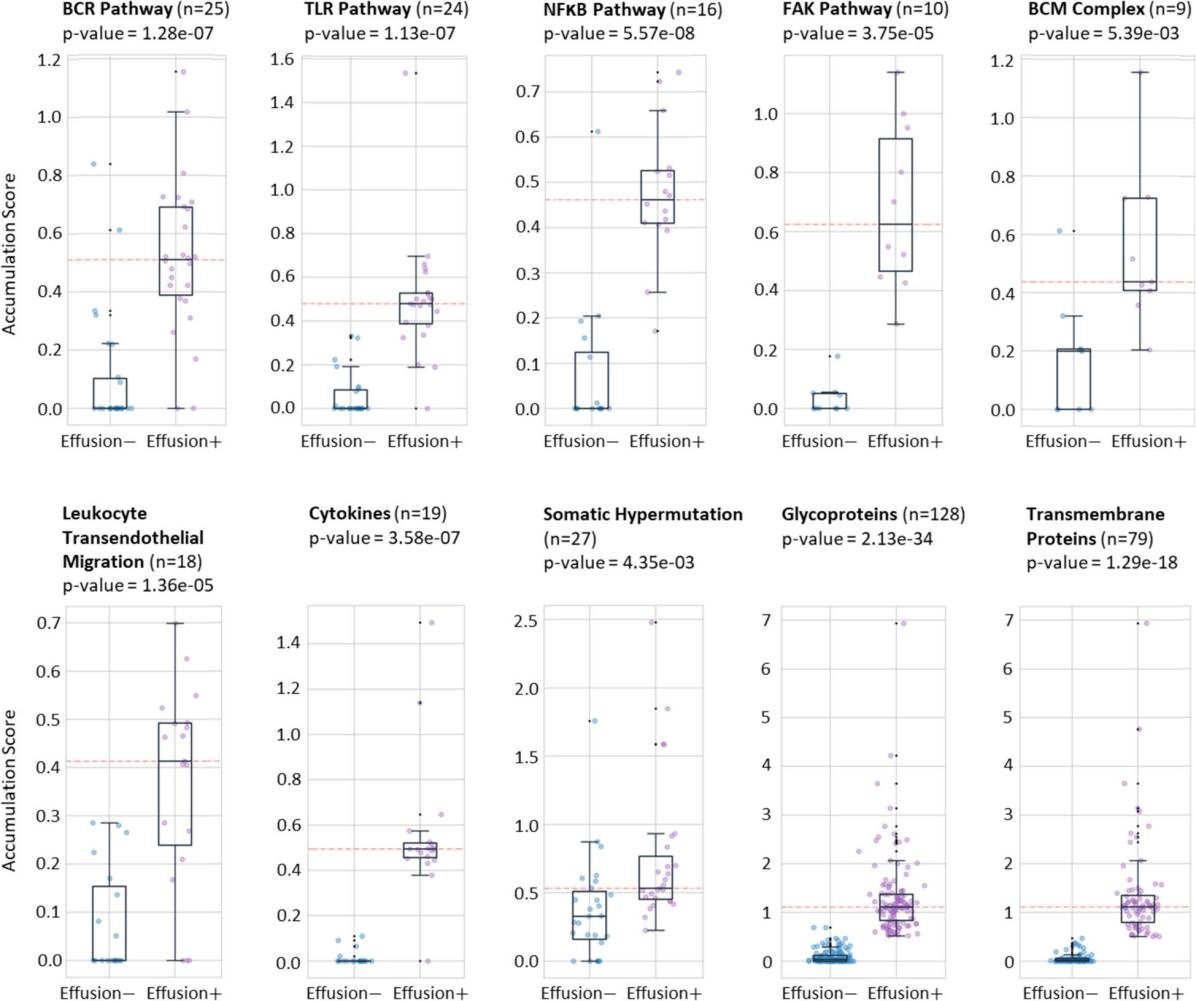
The comparison of the accumulation scores obtained for different migration/invasion-associated pathways and molecular groups in the two cohorts: effusion-associated (effusion+) DLBCL and non-effusion-associated (effusion—) DLBCL using boxplots. The accumulation scores for the migration/invasion-associated genes of the samples with tumors in effusions (> 0.4) are much higher than those bearing tumors in solid organs such as lymph nodes (< 0.2)

Next, we compared the accumulation score of the migration/invasion regulator genes in the above pathways and groups between the two DLBCL cohorts (with versus without lymphomatous effusion). As shown in Fig. 1, the boxplots compare the accumulation scores of the two cohorts for different pathways and groups. Interestingly, the genes in boxplots for the effusion-associated DLBCL cases showed significant higher accumulation scores compared to those of non-effusion-associated DLBCL cases. The mean score in the effusion-associated DLBCL dataset was higher than 0.4. By contrast, the mean value of the accumulation score in the non-effusion DLBCL dataset was less than 0.2 in most cases. The Supplementary Table S6 Excel file contains the accumulation scores of all genes in both datasets.

### Identification of the pathogenic and mutated genes in effusion-based DLBCL samples

**Fig. 2:**
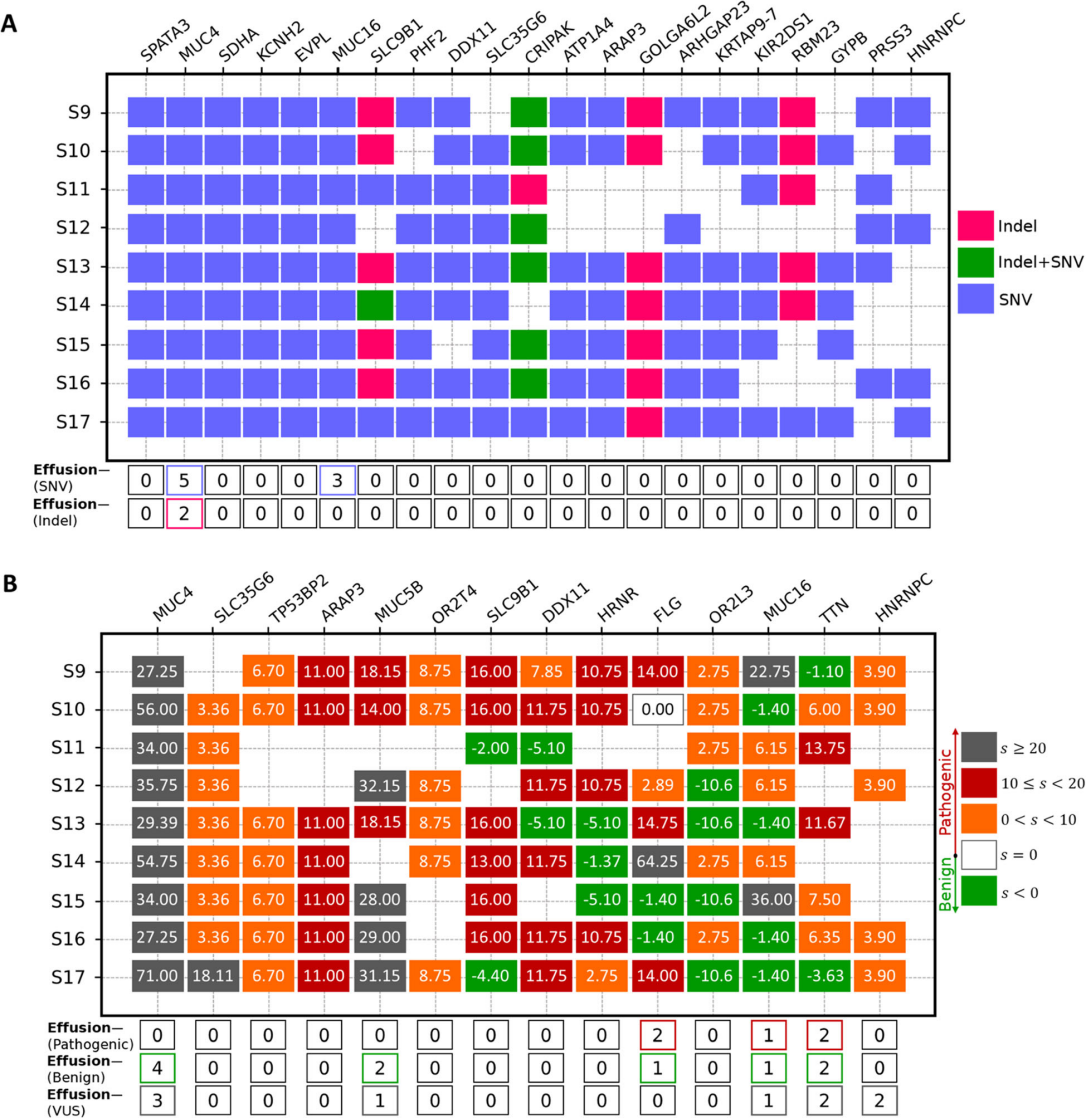
Genes affected by mutations in at least five effusion-associated (effusion+) DLBCL samples. (**A**) The samples containing at least one insertion/deletion (Indel) mutation, SNV, or both indel/SNV are represented by pink, blue, and green squares, respectively. The bottom of the figure shows the number of non-effusion-associated (effusion—) samples that contain SNVs and Indels. (**B**) Genes that have pathogenic mutation(s) in at least five effusion-associated (effusion+) DLBCL samples. Genes containing benign mutation(s) are shown by green (s < 0). Genes that have no pathogenic mutation and at least one variant of uncertain significance (VUS) are shown by white (s = 0). Genes that consist of at least one pathogenic mutation are shown by orange (0 < s < 10), red (10 < = s <= 20), or gray (s > 20) depending on the number of pathogenic mutations. The more positive score is interpreted as more pathogenic

Mutations and SNVs are rich sources for detecting the biomarkers of various diseases. Here, we considered mutations/SNVs of the samples with tumors in effusions. The details of the mutations/SNVs of the effusion-associated DLBCL dataset are listed in Supplementary Figures S3-S6. We obtained the genes affected by SNVs, indels, or both in at least five samples (Fig. 2 A). Supplementary Figure S7 shows the stacked bar charts of SNVs and indels in the effusion-associated and nodal-based DLBCL cohorts, and Supplementary Figure S8 comprises all genes affected by splice, indels, missense, multiple hits, stop-loss, and stop-gain mutations in at least one effusion-associated DLBCL sample. Although Fig. 2 A and Supplementary Figure S8 contain helpful information about the genes with their characteristics, there is no connection between the number of mutations in a gene and the malignant potential of an abnormal gene. For this reason, we extracted the genes that were detected as pathogenic in at least five effusion-associated DLBCL samples (Fig. 2B). As seen in Fig. 2, *MUC4* (pathogenic in 9/9 samples), *SLC35G6* (8/9), *ARAP3* (7/9), *SLC9B1* (6/9), *DDX11* (6/9), *MUC16* (5/9), and *HNRNPC* (5/9) are reported in both pathogenic and the mutated genes lists. Surprisingly, all genes that are reported in the two figures are mutated or pathogenic in a small number of the non-effusion-associated DLBCL samples except for *MUC4*, which was also frequently represented in non-effusion-associated DLBCL and warranted further validation.

### Gene set enrichment and pathway analyses

To decipher the gene expression profiling (GEP) responsible for lymphoma seeding in effusions, we additionally used cDNA microarrays to search for differentially expressed genes between effusion-associated DLBCL cases versus DLBCL without effusion. Since both groups of DLBCL cells were from the sites with different microenvironments, that is, one was embedded in solid matrix and the other floated in effusions, we carefully excluded genes engaged in microenvironment and matrix remodeling to minimalize the bias. As shown in Supplementary Table S7 Excel file, the genes with 1.5-fold changes of expression intensity in the effusion group were consequently subjected to the statistical simulations and analysis using Ingenuity Pathway Analysis (IPA) and Gene Set Enrichment Analysis (GSEA) software to identify canonical pathways.

**Fig. 3:**
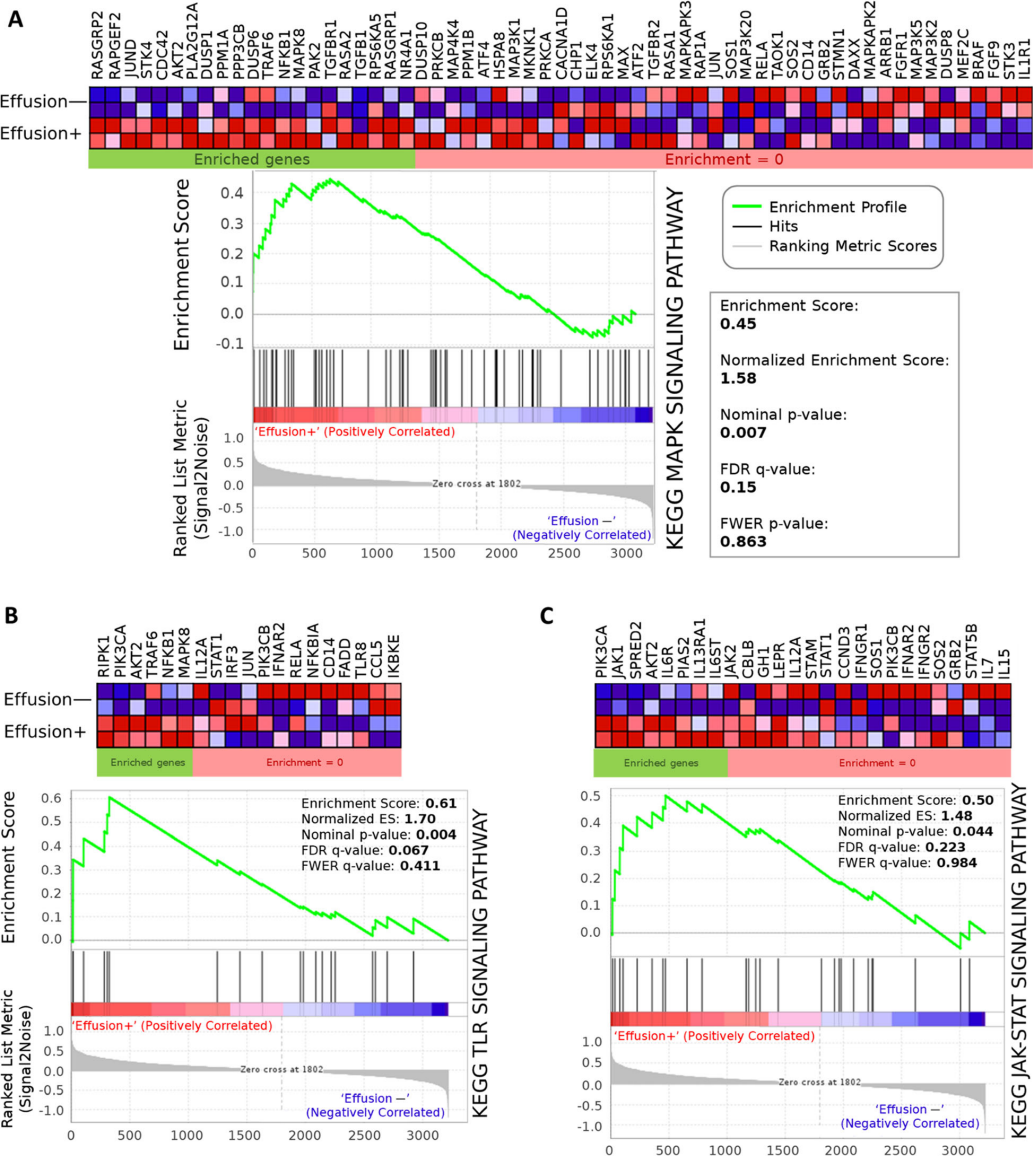
Gene set enrichment analysis (GSEA) on the gene expression data between effusion-associated DLBCL (effusion+) cases versus DLBCL without effusion (effusion—) cases. The results on KEGG gene set show that effusion-associated DLBCL is positively associated with the (**A**) MAPK signaling pathway, (**B**) TLR signaling pathway, and (**C**) JAK-STAT signaling pathway

**Fig. 4:**
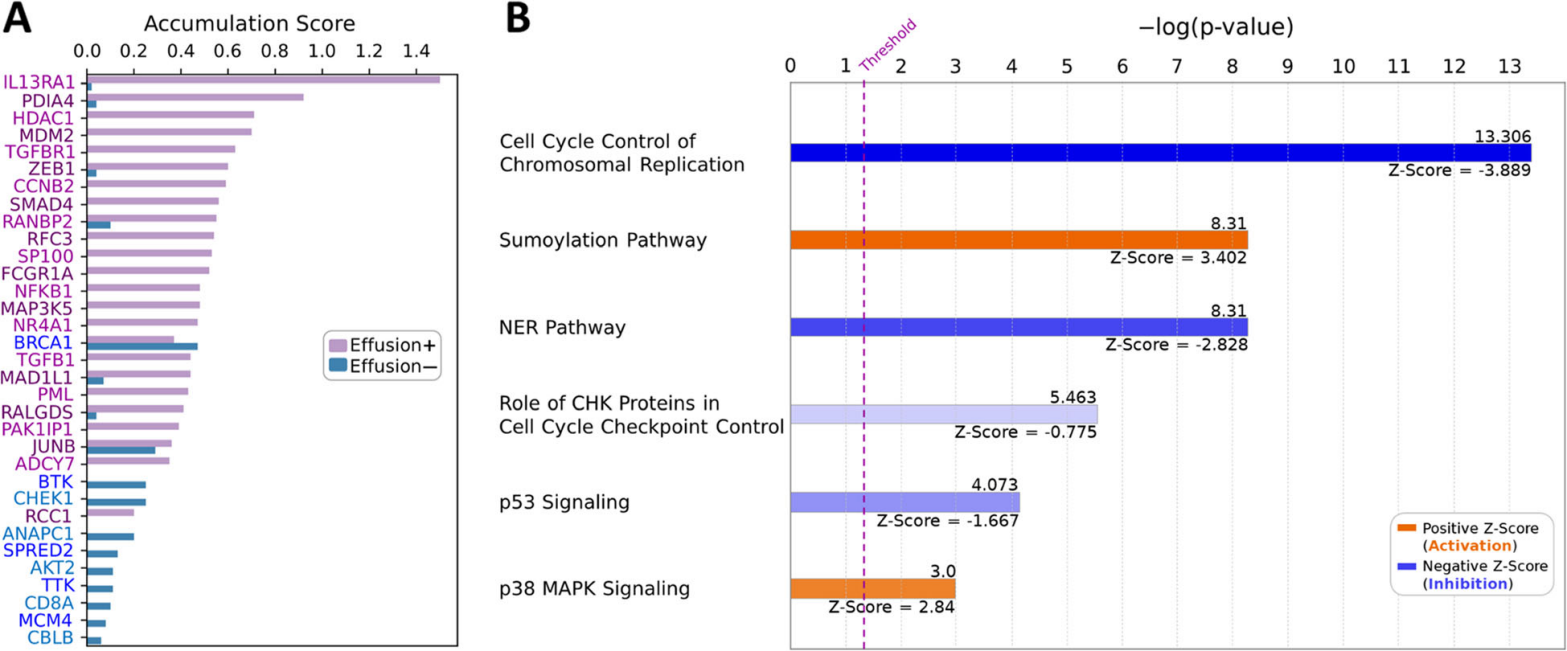
The accumulation scores of the partner proteins involve in the different pathways identified by IPA and GSEA. (**A**) The proteins with a high score in the effusion-associated (effusion+) cohort and non-effusion-associated (effusion—) cohort are represented by purple and blue, respectively. (**B**) The pathways that are positively and negatively associated with effusion-associated DLBCL detected by IPA. We highlighted the six maximum and minimum z-score values. The orange bars (positive z-score) indicate the activation of pathways, and the blue bars (negative z-score) indicate the inhibition of pathways

GSEA analysis showed that chronic myeloid leukemia (enrichment score (ES) = 0.55; p-value = 0.010), cell cycle (ES = 0.53; p-value = 0.000), MAPK (ES = 0.45; p-value = 0.007), WNT (ES = 0.57; p-value = 0.006), TGFβ (ES = 0.56; p-value = 0.014), VEGF (ES = 0.80; p-value = 0.000), TLR (ES = 0.61; p-value = 0.004), and JAK-STAT (ES = 0.50; p-value = 0.044) pathways were significantly activated in samples of DLBCL associated with lymphomatous effusions (Fig. 3 and Supplementary Figures S9). In addition, the single-sample analysis (ssGSEA) and Gene Set Variation Analysis (GSVA) calculating sample-wise gene set enrichment showed results consistent with GSEA findings (Table 3). On the other hand, IPA revealed that samples bearing tumor in effusions were significantly associated with pathways involving p53 signaling, nucleotide excision repair (NER), checkpoint kinase (CHK) proteins in cell cycle, DNA replication, sumoylation (SUMO), and p38 MAPK pathways. The details of each pathway extracted by IPA are shown in Supplementary Table S8 Excel file. We extracted the partner proteins involved in the pathways identified by IPA and GSEA. Figure 4 A illustrates the mutation accumulation scores of these proteins, which were also present in the NGS dataset. In this figure, we excluded the proteins in which their scores in both effusion-associated and non-effusion DLBCL groups were zero. The proteins with a higher score in the effusion-associated cohort compared to the non-effusion-associated cohort were represented by the purple color. In contrast, the blue-colored proteins had a higher score in the non-effusion-associated DLBCL cohort. As seen in Fig. 4 A, most of the genes identified by GSEA and IPA had higher accumulation scores in the effusion-associated DLBCL cohort, that is, the genes in Fig. 4 A were present in both microarray and NGS datasets and showed higher mutation scores in effusion-associated DLBCL cohort. In Fig. 4B, we highlighted the six pathways most associated with highest/lowest z-score values. The positive and negative z-score values indicate the activation and the inhibition of pathways, respectively, in the effusion-associated DLBCL cohort. Accordingly, we found activation of SUMOylation and p38 MAPK pathways (Supplementary Figures S10-S11) as well as inhibition/downregulation in cell cycle control of DAN replication, NER, cell cycle checkpoint control, and p53 signaling pathways (Supplementary Figures S12-S15).

**Table 3:** Results of Gene Set Variation Analysis (GSVA) and single-sample GSEA (ssGSEA)

	GSVA				ssGSEA			
Signaling pathway	E(+)1	E(+)2	E(-)1	E(-)2	E(+)1	E(+)2	E(-)1	E(-)2
MAPK	0.158	0.312	-0.236	-0.244	0.175	0.170	0.061	0.059
Toll-like receptor	0.160	0.168	-0.142	-0.177	0.170	0.177	0.113	0.116
JAK/STAT	0.359	0.208	-0.014	-0.498	0.083	0.076	-0.043	-0.059

Both GSVA and ssGSEA were implemented using “GSVA” package in R

### Validation of mutations in selected genes in cell lines and clinical samples of DLBCL

**Fig. 5:**
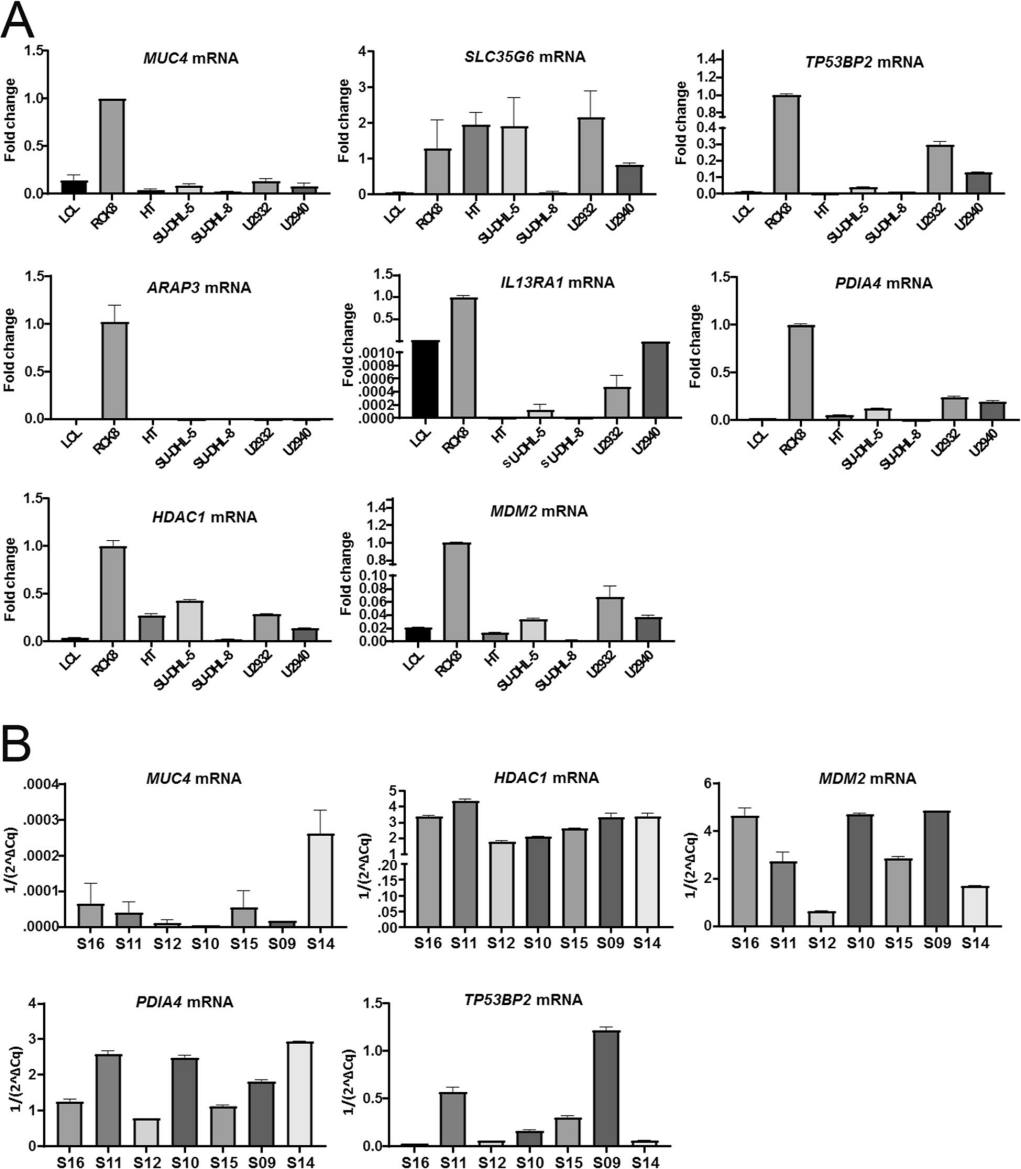
Validation of mRNA expression level of the interested genes in DLBCL cell lines and clinical samples. (**A**) Relative mRNA expression of 8 selected genes (*MUC4*, *SLC35G6*, *TP53BP2*, *ARAP3*, *IL13RA1*, *PDIA4*, *HDAC1* and *MDM2*) was measured in different DLBCL cell lines by quantitative reverse transcription PCR (RT-qPCR) (LCL cells as control). (**B**) The levels of *MUC4*, *HDAC1*, *MDM2*, *PDIA4*, *TP53BP2* mRNA were analyzed by RT-qPCR. *β-actin* gene expression was used as endogenous control. Error bars indicate the standard error of the mean in triplicate

**Fig. 6:**
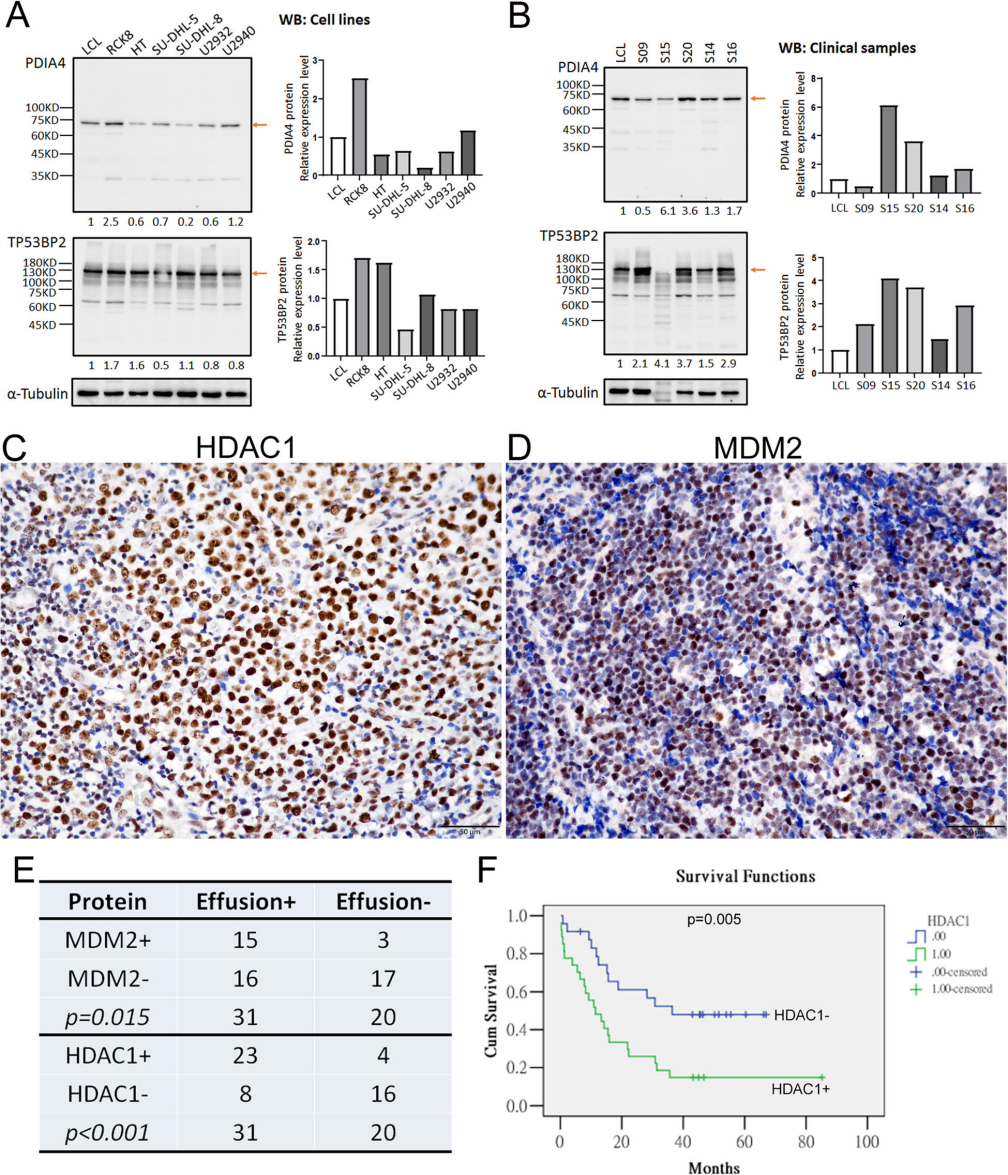
Validation of PDIA4, TP53BP2, HDAC1 and MDM2 protein levels in DLBCL cell lines and clinical samples. The protein level of PDIA4, TP53BP2 was evaluated (**A**) in various DLBCL cell lines and (**B**) clinical specimens by western blot (LCL cells as control). (**C**, **D**) Representative IHC staining of HDAC1 and MDM2 in an independent cohort of 51 DLBCL cases with (n = 31) and without (n = 20) lymphomatous effusions, respectively. Scale bars denote 50 μm. (**E**) The positive correlation between lymphomatous effusions and HDAC1 and MDM2 expression. (**F**) Kaplan-Meier survival analysis to compare the cumulative survival rate of all patients with various HDAC1 expression levels

We validated the expression of relevant genes yielded by NGS (Fig. 2B) and GEP (Fig. 4B) analyses in different cell lines and clinical samples of DLBCL. As shown in Fig. 5 A, quantitative reverse transcription PCR (qRT-PCR) confirmed the higher expression of *SLC35G6, MUC4, TP53BP2, PDIA4, HDAC1* and *MDM2* in most DLBCL cell lines compared with LCL (lymphoblastoid cell lines). The mRNA levels of *HDAC1, MDM2*, and *PDIA4* were highly expressed in most clinical samples in comparison with β-actin (Fig. 5B). Western blot (WB) analysis also confirmed that the protein levels of PDIA4 and TP53BP2 were highly expressed in most DLBCL cell lines (Fig. 6 A) and clinical samples (Fig. 6B). The lack of available SLC35G6 antibody prevented the validation of protein expression for this marker (https://www.antibodypedia.com/gene/82013/SLC35G6).

An independent cohort of 51 DLBCL cases (Supplementary Table S2) with (n = 31) and without (n = 20) lymphomatous effusions were immunohistochemically studied to validate the role of MUM4, HDAC1 and MDM2 overexpression in the formation of lymphomatous effusions. We found that overexpression of HDAC1 (Fig. 6 C) and MDM2 (Fig. 6D) correlated with the presence of lymphomatous effusions (Fig. 6E), and HDAC1 overexpression also was associated with a poorer prognosis (p = 0.005, Fig. 6 F). The expression of HDAC1 and MDM2 in non-effusion DLBCL cases and survival analyses for other clinicopathologic factors are shown in Supplementary Figure S16. Notably, although MUC4 was found to be pathogenic in the NGS analysis, all 51 DLBCL cases were negative for MUC4 expression.

## Discussion

In this study, we designed a method that assigned an RMD-based score to each gene across a population. We selected the genes that had high score in the cases of DLBCL associated with lymphomatous effusions and a low score (≈ 0) in the nodal-based DLBCL cases without effusions. Furthermore, we utilized four different mutation interpreters to identify pathogenic genes. The genes that were reported as pathogenic in most effusion-associated DLBCL samples but were benign in the nodal-based DLBCL samples without effusions might be associated with effusion-based DLBCL. Interestingly, we found that the genes with high RMD-based or pathogenicity scores were related to tumor invasion, migration, and adhesion. Finally, we validated the role of TP53-related and chromatin remodeling pathways in pathogenesis of DLBCL lymphomatous effusions in an independent cohort. We found that DLBCL-associated lymphomatous effusions may be associated mechanistically with TP53-MDM2 pathway and HDAC-related chromatin remodeling mechanisms.

It is also interesting that the effusion cases harbor much more Indels and SNVs than the non-effusion cases (Supplementary Figure S7). These findings are not well-known. The non-effusion group from TCGA was nodal-based and analyzed by using Mutect2 to call somatic variants in tumor and matched normal samples. Variants with MAF > 1 % in public and in-house databases (gnomAD East Asian and Taiwan biobank) were excluded (filtered) for further analysis. In contrast, for the effusion-positive group, we used Freebayes to call potentially somatic variants in tumor samples, considered only exonic or splicing variants, and removed synonymous SNV. Thus, the possible causes for that the effusion cases harbor much more Indels and SNVs than the non-effusion cases are described as follows: first, the filter strategy: TCGA group first used matched normal tissues to subtract potential candidate genes but we did not; second, the tumor cell biology: spreading (metastatic) tumor cells acquire additional mutated genes to accomplish the dissemination process than primary (lymph node) tumor cells. So, it is reasonable that the effusion cohort (metastatic tumors) carries much more mutated genes than the non-effusion cohort (primary tumors).

Focusing on the connection with migration/invasion pathways is important. Thus, we utilized various migration/invasion-associated pathways to calculate the mutation accumulation scores of their regulator genes in DLBCL with or without lymphomatous effusions. We found the following pathways more highly activated in the effusion-associated DLBCL cohort, including BCR, NFκB, TLR, FAK, BCM complex, cytokines, and glycoproteins. Extracellular stimuli activate and phosphorylate the MEKK1-MKK4 (or MKK7)-JNK-FAK signaling through FAK (PTK2); next, activated JNK phosphorylates Jun, Paxillin, or Spir [25], and phosphorylated Jun promotes cell migration. Phosphorylation of Paxillin accelerates turnover of cell adhesion, and promotes rapid cell motility [25]. The phosphorylated Spir also affects actin dynamics and cell migration. Furthermore, FGF and EGF growth factors activate FAK via the Ras-Raf-MEK-Erk-FAK signaling module [25].

One of the major regulators of lymphocyte survival, proliferation, and activation is the transcription factor NFκB that provides association between chronic inflammation and lymphomagenesis. NFκB activation increases the production of anti-apoptotic factors and chemokines that trigger the migration of immune cells to inflammatory foci. These properties promote tumor cell survival and metastasis, while inhibit apoptosis [26]. The BCL10-CARD11-MALT1 (BCM) complex plays a key role in forming an essential connection between NFκB activation and the triggering of cell surface antigen receptors. Genetic and biochemical methods show that the connection between the BCM complex and activated NFκB functionally leads to migration and invasion [27]. The pathways that are involved in activating NFκB signaling can promote tumor growth. The interaction of pathogens with Toll-like receptors (TLRs) on the cell surface is known to activate the NFκB pathway. The activation of NFκB results in upregulation of interleukin (IL)-10, IL-16, IL-2, IL-1, and increased production of various pro-inflammatory cytokines [28]. The activation of TLR signaling in tumor cells stimulates metastasis and enhances the proliferation of cancer cells via angiogenic factors, such as MMP, VEGF, and IL-8 [29]. Inflammatory mediators such as cytokines can suppress the DNA mismatch repair system across various mechanisms, which subsequently result in genetic mutations [30].

Signaling through the BCR activates the cytoplasmic domain of integrin, causing a conformational alteration in the extracellular domain that induces cell migration [31]. The accumulation of somatic mutations in essential genes of the BCR pathway highlights the key role of BCR signaling in DLBCL tumorigenesis. Most *CD79A/B* mutations result in deletions of large segments of the immunoreceptor tyrosine-based activation motif (ITAM) region [32]. These mutations have been shown to enhance BCR surface expression levels [32]. Glycosylation affects tumor growth and survival and promotes metastasis [33]. Aberrant glycosylation in tumors is linked with oncogenic transformation and plays a crucial role in progression, growth, and metastasis [34, 35]. Oliveira-Ferrer et al. [35] have shown that glycoproteins influence distinct stages of the metastatic process, such as migration and invasion of cancer cells.

We also highlighted the genes affected by mutations or detected as pathogenic in at least five effusion-associated DLBCL samples. Here, we discuss the connection between some of the genes with migration/invasion of cells. MUC4 facilitates metastases by promoting a group of tumor cells that diffuse into the bloodstream. A study has shown that MUC4 intensifies invasion and migration potential and promotes metastasis and oncogenesis [36]. MUC16 increases the invasion, migration, and proliferation of cancer cells in vitro, and also enhances tumorigenesis and metastasis in vivo [37]. ARAP3, which is found in the plasma membrane, is essential for lamellipodia formation after stimulation of the growth factor signaling on activation of PI3K pathway [38]. Wang et al. identified that downregulation of ARAP3 significantly inhibited the invasive and migratory abilities of thyroid cancer cell lines [39]. TP53BP2 (ASPP2) inhibition accelerates cell migration, invasion, and epithelial-mesenchymal transition in breast cancer cells [40]. Notably, although MUC4 was found to be pathogenic in the NGS analysis, all 51 DLBCL cases were negative for MUC4 expression. These data highlight the importance of validating protein expression on clinical samples. Alternatively, it is possible that MUC4 mutations lead to loss of function.

GSEA analysis revealed that a set of pathways such as WNT, VEGF, and JAK-STAT were significantly activated in samples of DLBCL-associated lymphomatous effusions. Aberrant activation of the WNT pathway promotes abnormal cellular behaviors such as cell motility, matrix invasion, and tumor progression [41]. Inhibition of VEGF signaling in colon cancer cell lines strongly inhibits cancer cell migration and invasion by regulatory proteins associated with cell motility [42]. JAK1 is the main activator of STAT3 in many cellular systems [43]. An earlier study has shown that the JAK-STAT signaling largely correlates with the invasion and migration [44]. Increased STAT3 expression has been reported in metastases at the leading edges of invasive cancers [45].

IPA analysis identified p53 signaling, nucleotide excision repair (NER), checkpoint kinase (CHK) proteins in cell cycle, DNA replication, sumoylation (SUMO), and p38 MAPK pathways that were significantly associated with samples bearing tumor in effusions. p53 is a tumor suppressor whose loss perturbs cell-cycle checkpoints. p53 inhibition also devastates pathways that reduce metastasis. p53 directly controls the transcription of genes that are involved in cell adhesion, motility, and invasion [46]. Dysregulation of p38 is associated with metastases and low survival rates [47]. Furthermore, a well-known anti-cancer drug, baicalein, inhibits cancer cell motility and metastasis through inhibition of the p38 signaling pathway [48]. The NER signaling pathway identifies and removes a wide variety of DNA damage. Aberrations in NER-associated genes have been illustrated in several malignancies with a potential impact on clinical issues [49]. The Ranbp2 protein relates to cancer cells, and genetic point mutations and translocations are associated with tumorigenesis [50]. Interaction of insulin-like growth factor-1 receptor (IGF1R) with Ranbp2 is essential for IGF1R sumoylation that plays a crucial role in cancer cell progression [51]. On the other hand, some polymorphisms of the SUMO-conjugating enzyme ubc9 are associated with metastasis and invasion [52]. Furthermore, an increase in AP-1 expression promotes the migration, invasion, and metastasis [53]. DNA must be replicated precisely before cell division happens. Defective DNA replication triggers aberrant types of DNA replication, such as DNA re-replication and unscheduled endoreplication, which leads to more aggressive and drug-resistant forms of cancer. The kinases ataxia telangiectasia and rad3-related protein (ATR), ataxia telangiectasia mutated (ATM), and checkpoint kinase 1/2 (CHK1/2) form a crucial DNA damage response module at the stalled replication fork, which is recognized as replication stress. The activation of ATM-ATR phosphorylates CHK1 and CHK2, which further activates p53 and other downstream molecules as well as the response proteins to replication stress [54].

The GSEA and IPA analysis revealed 23 enriched genes including *PDIA4*, *HDAC1*, and *MDM2* with higher mutation accumulation scores in the effusion-associated DLBCL samples versus the comparison cohort. PDIA4 has been reported to act as a promoter of tissue factor responsible for coagulation, modulating the function and accumulation of platelets [55]. Stimulated platelets assist in promoting tumor cell metastasis, proliferation, adhesion, and angiogenesis and keep the tumor cells away from the immune system [56]. Thus, PDIA4 might be connection between activated platelets and tumor progression [57]. PDIA2 is a critical prognostic marker that plays an important role in the drug-resistance phenotype in ovarian carcinoma [58]. High expression levels of HDAC1 have been reported in diverse cancer types. The expression level of some histone-related proteins including HDAC1, HDAC2, and HDAC6 was significantly higher in cases of DLBCL compared to normal lymphoid tissue. In addition, increased expression of HDAC1 was related to the tumor aggressiveness and a poorer survival in patients with DLBCL [59]. In parallel, we found that overexpression of HDAC1 and MDM2 correlated with the emergence of lymphomatous effusions, and HDAC1 overexpression further predicted worse outcome. The p53 tumor suppressor is negatively regulated by MDM2. p53 transactivates *MDM2*, and then MDM2, in turn, degrades and inhibits p53 activity forming a negative feedback loop. MDM2 is reported to be highly expressed in cancers [60]. A noteworthy strategy to activating p53-mediated apoptosis in tumors, which overexpress MDM2 and wild-type p53, is to inhibit the interaction of the p53-MDM2 [61]. Our findings suggest that DLBCL-associated lymphomatous effusions may be associated mechanistically with TP53-MDM2 pathway and HDAC-related chromatin remodeling mechanisms.

The weakness of our study is a relatively small number of samples, which is due to the rare occurrence of DLBCL presenting initially with lymphomatous effusions. However, we used two methods, WES and gene microarray, as well as delicate bioinformatics analyses to decipher the genes responsible for the pathogenesis of lymphomatous effusions in DLBCL. Furthermore, we validated our NGS findings with an additional cohort of clinical samples with or without tumorous effusions and confirmed the interesting findings, which warrant further studies with larger, independent cohorts.

## Conclusions

DLBCL is the most common type of lymphoma. A subset of patients may present with lymphomatous effusions initially or during disease progression, and is associated with a poor prognosis of affected patients [4]. In this study we used NGS and GEP to assess effusion-based DLBCL cells and compared the results to non-effusion-associated DLBCL cells. The results showed that effusion-associated DLBCL cells got higher scores in the BCR, NFκB and TLR pathways and in genes responsible for leukocyte migration. On the other hand, IPA highlighted the pivotal role of TP53-related and chromatin remodeling pathways in DLBCL lymphomatous effusions, which were validated on an independent cohort. Our findings have shed light on the prognostic and therapeutic implications for DLBCL patients with lymphomatous effusions.

## Supplementary Information

**Additional file 1.**

**Additional file 2.**

**Additional file 3.**

**Additional file 4.**

**Additional file 5.**

**Additional file 6.**

## Data Availability

The data that support the findings of this study are available on request from the corresponding author. WES data are available in the National Center for Biotechnology Information (https://submit.ncbi.nlm.nih.gov/).

## Abbreviations

ATM: ataxia telangiectasia mutated
ATR: ataxia telangiectasia and rad3-related protein
BCM: BCL10-CARD11-MALT1
BCR: B-cell receptor
CHK: checkpoint kinase
DLBCL: diffuse large B-cell lymphoma
ES: enrichment score
FAK: focal adhesion kinase
GEP: gene expression profilin
GSEA: Gene Set Enrichment Analysis
ssGSEA: single-sample GSEA
GSVA: Gene Set Variation Analysis
IL: interleukin
IPA: Ingenuity Pathway Analysis
ITAM: immunoreceptor tyrosine-based activation motif
LCL: lymphoblastoid cell lines
MAF: minor allele frequency
NER: nucleotide excision repair
NGS: next generation sequencing
RMD: regional mutation density
qRT-PCR: quantitative reverse transcription PCR
SNV: single nucleotide variant; indels, insertions/deletions
SUMO: sumoylation
TCGA: The Cancer Genome Atlas
TLR: toll-like receptors
WES: whole exome sequencing
WB: Western blot
