# Supplementary material for: Deciphering genes associated with diffuse large B-cell lymphoma with lymphomatous effusions: A mutational accumulation scoring approach
Source: Biomark Res. 2021 Oct 9;9:74. doi: 10.1186/s40364-021-00330-8 (PMC8504051; doi:10.1186/s40364-021-00330-8)
Supplement: Supplementary file 1 — Additional file 1. [file 40364_2021_330_MOESM1_ESM.pdf]

Supplementary Information

Deciphering genes associated with diffuse large B-cell lymphoma with lymphomatous effusions: A mutational accumulation scoring approach

Sina Abdollahi, Seyedeh Zahra Dehghanian, Liang-Yi Hung, Shiang-Jie Yang, Dao-Peng Chen, L. Jeffrey Medeiros, Jung-Hsien Chiang, and Kung-Chao Chang

Summary

Supplementary Materials and Methods

Supplementary References

Supplementary Tables S1-S8 (Supplementary Tables S4-S8 in Excel files)

- Supplementary Tables S4 WES
- Supplementary Tables S5 Boxes genes score in different pathway
- Supplementary Tables S6 Genes score Effusion(+) and Effusion(-)
- Supplementary Tables S7 Expression for GSEA
- Supplementary Tables S8 IPA analysis results

Supplementary Figures S1-S16

Supplementary Materials and Methods

Bioinformatics analyses

Raw reads from fastq were trimmed for adaptor sequence and reads with low quality using trimmomatic version 0.36 [1]. The qualified reads were mapped to the human reference genome GRCh37 using BWA-MEM version 0.7.13 [2]. Following alignment, the resulting BAM files were sorted and removed duplicate reads using PICARD (http://broadinstitute.github.io/picard/). Freebayes version 1.2.0 [3] was used to call single nucleotide variants (SNVs) and indel with default settings. Variants in VCF were filtered out if they fulfilled one of the following criteria: 1) QUAL < 20; 2) Taiwan Biobank minor allele frequency (MAF) > 1%, 3) gnomAD East Asian MAF > 1%, 4) the total number of reads at the position < 20, 5) the total number of reads at the position with mutant alleles < 10, and 6) variant allele frequency < 0.1.

Mutation interpreters

In this study, we utilized four following mutation interpreters for calculating the pathogenicity score of each mutation: (1) SIFT used sequence homology and physicochemical properties of an amino acid substitution and predicted its pathogenicity [4]; (2) CADD utilized various genome annotations from different databases and scored the pathogenicity of any SNV or indel [5]; (3) InterVar interpreted each mutation based on ACMG-AMP standards and guidelines [6]; (4) ClinVar recorded the association between mutations and observed clinical conditions [7].

References

1. Bolger AM, Lohse M, Usadel B. Trimmomatic: A flexible trimmer for Illumina sequence data. Bioinformatics 2014; 30: 2114–2120.

2. Li H. Aligning sequence reads, clone sequences and assembly contigs with BWA-MEM. 2013.http://arxiv.org/abs/1303.3997.

3. Garrison E, Marth G. Haplotype-based variant detection from short-read sequencing. 2012. http://arxiv.org/abs/1207.3907.

4. Sim NL, Kumar P, Hu J, Henikoff S, Schneider G, Ng PC. SIFT web server: Predicting effects of amino acid substitutions on proteins. Nucleic Acids Res 2012; 40: W452–W457.

5. Rentzsch P, Witten D, Cooper GM, Shendure J, Kircher M. CADD: Predicting the deleteriousness of variants throughout the human genome. Nucleic Acids Res 2019; 47: D886–D894.

6. Li Q, Wang K. InterVar: Clinical Interpretation of Genetic Variants by the 2015 ACMG-AMP Guidelines. Am J Hum Genet 2017; 100: 267–280.

7. Landrum MJ, Lee JM, Riley GR, Jang W, Rubinstein WS, Church DM et al. ClinVar: Public archive of relationships among sequence variation and human phenotype. Nucleic Acids Res 2014; 42: D980–D985.

8. An Q, Robins P, Lindahl T, Barnes DE. C→T mutagenesis and γ-radiation sensitivity due to deficiency in the Smug1 and Ung DNA glycosylases. EMBO J 2005; 24: 2205–2213.

9. Alsøe L, Sarno A, Carracedo S, Domanska D, Dingler F, Lirussi L et al. Uracil Accumulation and Mutagenesis Dominated by Cytosine Deamination in CpG Dinucleotides in Mice Lacking UNG and SMUG. Sci Rep 2017; 7. doi:10.1038/s41598-017-07314-5.

Supplementary Tables

Supplementary Table S1. Characterization of DLBCL cell lines and LCL

Cell line	Source	Subtype or immunophenotyping
HT	ATCC CRL-2260	Germinal center B cell (GCB)
SU-DHL-5	ATCC CRL-2958	Germinal center B cell (GCB)
SU-DHL-8	ATCC CRL-2561	Germinal center B cell (GCB)
HBL2	Gift from Dr. Chen YP	Germinal center B cell (GCB)
RC-K8	DSMZ ACC 561	Germinal center B cell (GCB)
U2932	DSMZ ACC 633	Activated B cell (ABC)
U2940	DSMZ ACC 634	Primary mediastinal large B-cell lymphoma
LCL	Gift from Dr. Chang Y	EBV-transformed B lymphoblast

All are negative for Epstein-Barr virus (EBV). The authentication of cell lines was performed by short-tandem repeat profiling, and Mycoplasma testing was done by conventional PCR methods.

Supplementary Table S2. The validation cohort of DLBCL patients with or without lymphomatous effusions

Case	Sex	Age	LDH	IPI score	Stage	MDM2	HDAC1	Effusion	Treatment	Survival	Months
1	F	66	483	3	4	-	-	No	COP*1, R-COP*2, R-CHOP*5	Yes	60.4
2	M	67	153	1	2	-	-	No	R-CEOP*4, R/T 3420cGy	No	28.2
3	M	62	118	2	3	-	-	No	COP*1, R-COP*3, COP*3	No	36.4
4	F	71	252	2	2	-	+	No	R-CHOP*6	No	31.4
5	M	47	242	2	4	+	-	No	R-CHOP*6, the R/T 3000cGy	Yes	67.0
6	M	76	464	3	3	-	-	No	R-CHOP*6	Yes	55.5
7	F	68	328	3	1	-	+	No	R-CHOP*4, R-ESHAP*1	No	7.8
8	M	74	218	2	1	-	-	No	R-CHOP*6, R-ESHAP*6	No	30.8
9	F	59	475	2	1	-	-	No	Sx, CEOP*1, R-CEOP*5	Yes	50.2
10	M	88	125	2	3	-	-	No	R-CEOP*3, R qm*3, R/T 4500cGy	Yes	6.5
11	F	73	338	3	4	-	-	No	COP	No	0.8
12	F	27	293	2	4	-	+	No	R-CEOP*6, R-ESHAP*4, allo-PBSCT	Yes	85.2
13	F	58	133	0	2	-	-	No	CEOP*1, R-CEOP*3, R/T 3600 cGy	Yes	66.2
14	M	58	392	2	4	-	-	No	R-CEOP*6	Yes	51.6
15	F	60	177	2	4	-	-	No	R-CEOP*6	Yes	46.4
16	F	51	150	0	2	+	-	No	CEOP*1, R-CEOP*3, R-ESHAP*4	Yes	45.7
17	M	52	272	2	3	-	-	No	R-CEOP*6, R/T 4900cGy	Yes	45.3
18	F	13	145	0	2	+	+	No	NHL98B-K2-A1	Yes	45.0
19	F	63	241	3	3	-	-	No	CHOP*1, R-CEOP*5	Yes	43.0
20	F	57	823	3	4	-	-	No	CEOP*1, R-CEOP*5, R/T for brain metastasis	No	10.1
21	F	60	881	4	4	+	+	Pleural effusion / ascites	CHOP*1, R/T 4500cGy	No	30.9
22	F	37	NA	0	2	-	-	Pleural effusion	Sx, CEOP*6	Yes	54.0
23	F	70	450	4	3	+	+	Pleural effusion	Bleomycin+Oncovin*1, R*1)	No	1.2
24	M	69	629	5	4	-	-	Pleural effusion / ascites	CEOP*1, R-CEOP*5, R/T	No	9.3
25	M	44	745	2	4	-	-	Pleural effusion	Sx, CEOP*1, R-CEOP*5, R-ESHAP*5, CHOP*1, allo-PBSCT	No	18.8
26	F	64	1574	4	4	-	+	Pleural effusion	Solucortef only	No	0.5
27	M	74	174	4	4	-	-	Pleural / pericardial effusions	PAdriaCEBO*2	No	2.2
28	F	48	148	1	1	-	-	Pleural effusion	High dose MTX, R/T 5000cGy, ESHAP*2	No	15.2
29	M	72	552	4	4	-	-	Pleural effusion	CEOP*1, R-CEOP*5	No	15.6
30	M	82	NA	>=3	>=3	-	+	Pleural effusion	Supportive care	No	0.3
31	M	67	960	5	>=3	+	+	Pleural effusion	COP*1	No	0.8
32	F	67	307	5	4	+	+	Pleural effusion	R/T*20, R-CEOP*6, R*3	No	13.3

33	F	51	381	3	3	+	+	Pleural effusion	R/T, CEOP*1, R-CEOP*5, ESHAP*4	No	14.2
34	M	63	1425	4	>=3	+	+	Pleural effusion	Supportive care	No	3.9
35	M	30	206	>=2	NA	+	+	Ascites	Supportive care	No	0.2
36	F	71	659	3	2	+	+	Pleural effusion	COP*1, R-COP*3, R-CHOP*5, R/T	No	8.2
37	M	48	601	4	4	-	-	Pleural effusion	Omaya C/T MTX/ara-C, IT R/T	No	12.4
38	M	62	228	3	3	-	+	Pleural effusion	CEOP*7, ESHAP*6, m-BACOP*2	No	21.9
39	M	57	321	1	2	+	+	Pleural effusion	Hyper-CVAD*1, R-CEOP*6, R-ESHAP*5, R-MINE, auto-PBSCT	No	35.6
40	M	51	228	2	1	+	+	Pleural / pericardial effusions	Sx, R/T 4000cGy, R-CHOP*8, R/T 4500cGy, R-CEOP*6	Yes	46.7
41	M	70	234	2	2	+	+	Pleural effusion	R-CEOP*4	Yes	43.2
42	M	73	174	1	1	-	+	Pericardial effusion	CHOP*3, R/T	No	22.2
43	F	76	428	3	4	-	-	Pleural effusion	CHOP*6, R/T, Endoxan 1 qd, steroid	No	11.7
44	M	80	210	5	4	+	+	Pleural effusion	COP*1	No	1.3
45	M	45	236	3	3	-	+	Pleural effusion	CEOP*6, R	No	15.5
46	F	69	248	4	4	+	+	Pleural effusion	R-COP*8, VP-16+Slou-Medrol+Carboplatin+Ara-C*1	No	9.2
47	F	73	297	5	4	-	+	Pleural effusion	CEOP*4, R*3	No	5.5
48	M	64	386	2	2	-	+	Pleural effusion	CEOP*1, R-CEOP*5	No	6.4
49	F	19	205	3	4	-	+	Pleural effusion	CEOP*3, m-BACOD*4, ESHAP*2, R/T, ICE*4	No	10.9
50	M	60	218	4	3	+	+	Pleural effusion	R-CEOP*6, R/T	No	15.9
51	M	55	910	2	2	+	+	Pleural effusion	R-CEOP*6, IT with Ara-C, R-ESHAP*3, R/T	No	11.4

Abbreviations: LDH, lactate dehydrogenase; IPI score, international prognostic index; MDM2, murine double minute 2; HDAC1, histone deacetylase 1; PE, pleural effusion; A, ascites; PCE, pericardial effusion; +, positive; -, negative; R-COP (Rituximab, cyclophosphamide, vincristine, prednisone); R/T, Radiotherapy; CHOP (cyclophosphamide, doxorubicin, vincristine, prednisone); CEOP (cyclophosphamide, epirubicin, vincristine, prednisone); Sx, surgery; ESHAP (etoposide, methylprednisolone, high-dose cytarabine, cisplatin); PBSCT, peripheral blood stem cell transplantation; PAdriaCEBO (prednisolone, adriamycin/doxorubicin, cyclophosphamide, etoposide, bleomycin, oncovin/vincristine); MTX, methotrexate; IT, intrathecal; m-BACOD (methotrexate, bleomycin, doxorubicin, cyclophosphamide, vincristine and dexamethasone); MINE (mesna, ifosfamide, mitoxantrone, etoposide); Hyper-CVAD (cyclophosphamide, vincristine, doxorubicin, dexamethasone, methotrexate and cytarabine); ICE (ifosfamide, carboplatin, etoposide).

Supplementary Table S3. Whole exome sequencing (WES) information of 9 effusion-associated DLBCL samples

Case	Number of reads	Throughput (bp)	Mean depth in targeted region	Coverage in targeted region
S9	73756116	11137173516	276.67	99.87%
S10	91098664	13755898264	377.18	99.81%
S11	84794307	12803940357	354.07	99.87%
S12	70350619	10622943469	209.68	99.57%
S13	62914270	9500054770	197.408	99.64%
S14	71946504	10863922104	289.4	99.85%
S15	72607868	10963788068	301.919	99.67%
S16	80487216	12153569616	336.97	99.86%
S17	80275035	12121530285	336.845	99.66%

Supplementary Table S4 Excel file. Details of WES in 9 cases of effusion-associated DLBCL

Supplementary Table S5 Excel file. Essential genes in different pathways

Supplementary Table S6 Excel file. Accumulation scores of all genes in the effusion-associated DLBCL and non-effusion-associated DLBCL datasets

Supplementary Table S7 Excel file. Genes differentially expressed on lymphoma cells in solid organs versus in effusions

Supplementary Table S8 Excel file. Details of each pathway extracted by IPA

Supplementary Figures

Supplementary Figure S1. Gene scoring process

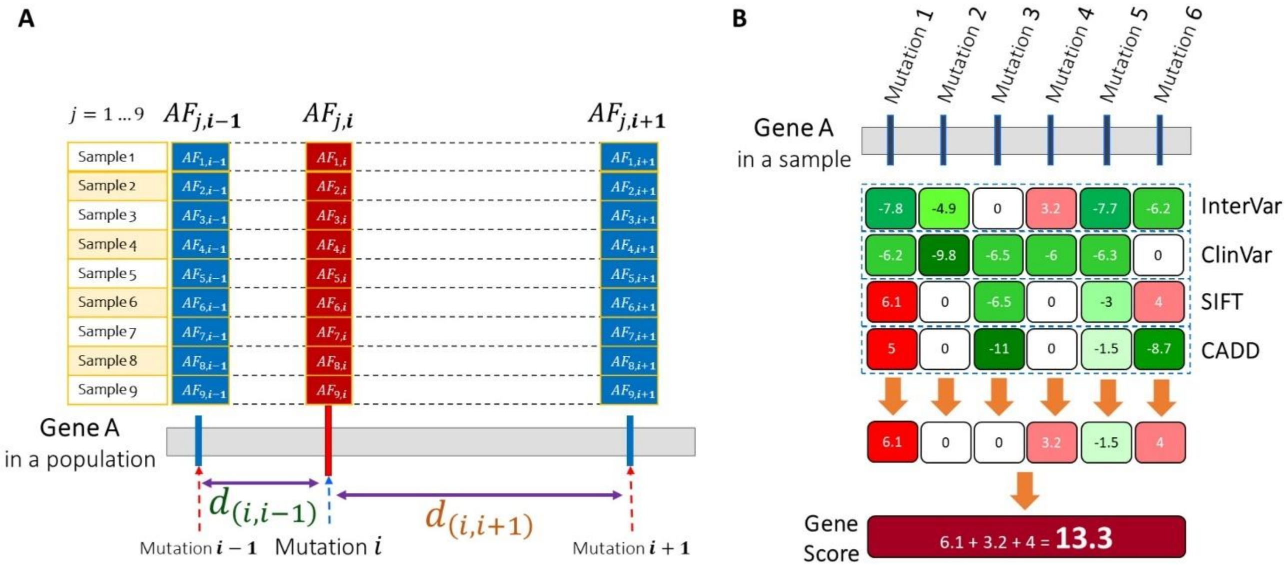

Supplementary Figure S1. (A) We assumed that all mutations of a population occur in a single nucleotide sequence. Hence, we maintained a list for each mutation that represents samples that contain the mutation. Each list encompasses the allele frequency in each sample of its corresponding mutation. Each mutation might occur in one or more samples. For each mutation i, we calculate its distance, in base pairs, with its vicinity mutations ($i-1$ and $i+1$). (B) A consensus method to obtain the pathogenicity score of each gene. The positive (red cells), negative (green cells), and zero scores (white cells) represent pathogenic, benign, and variant of uncertain significance, respectively.

Supplementary Figure S2. WES information and details of effusion-based DLBCL

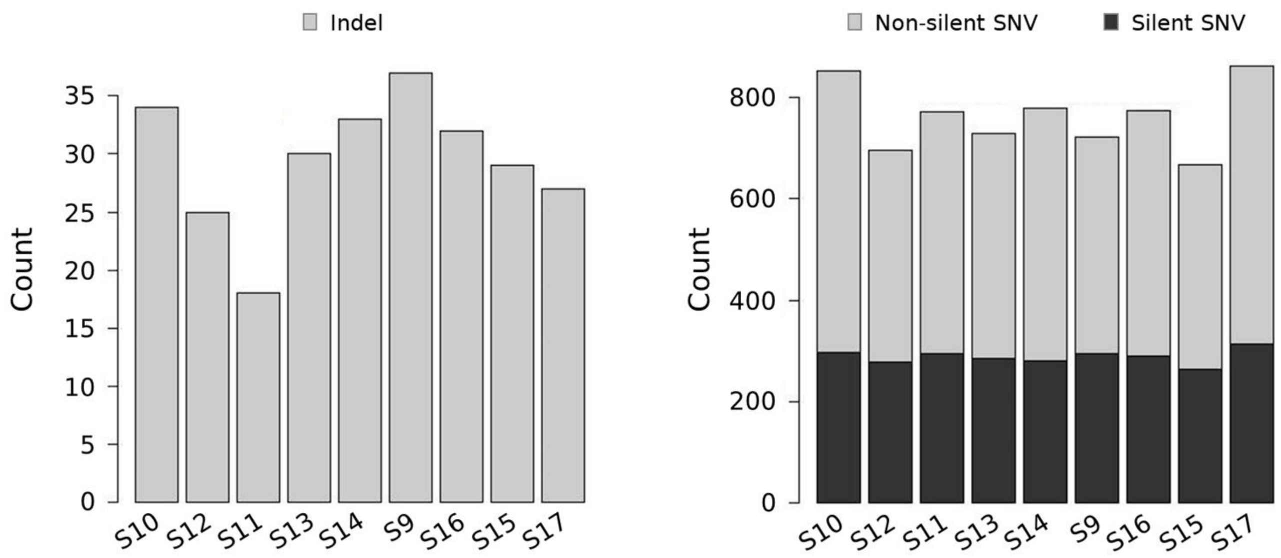

Supplementary Figure S2. The relative frequency of the silent and non-silent SNVs in nine samples of effusion-associated DLBCL.

Supplementary Figure S3. Number of mutations and single nucleotide variants (SNVs)

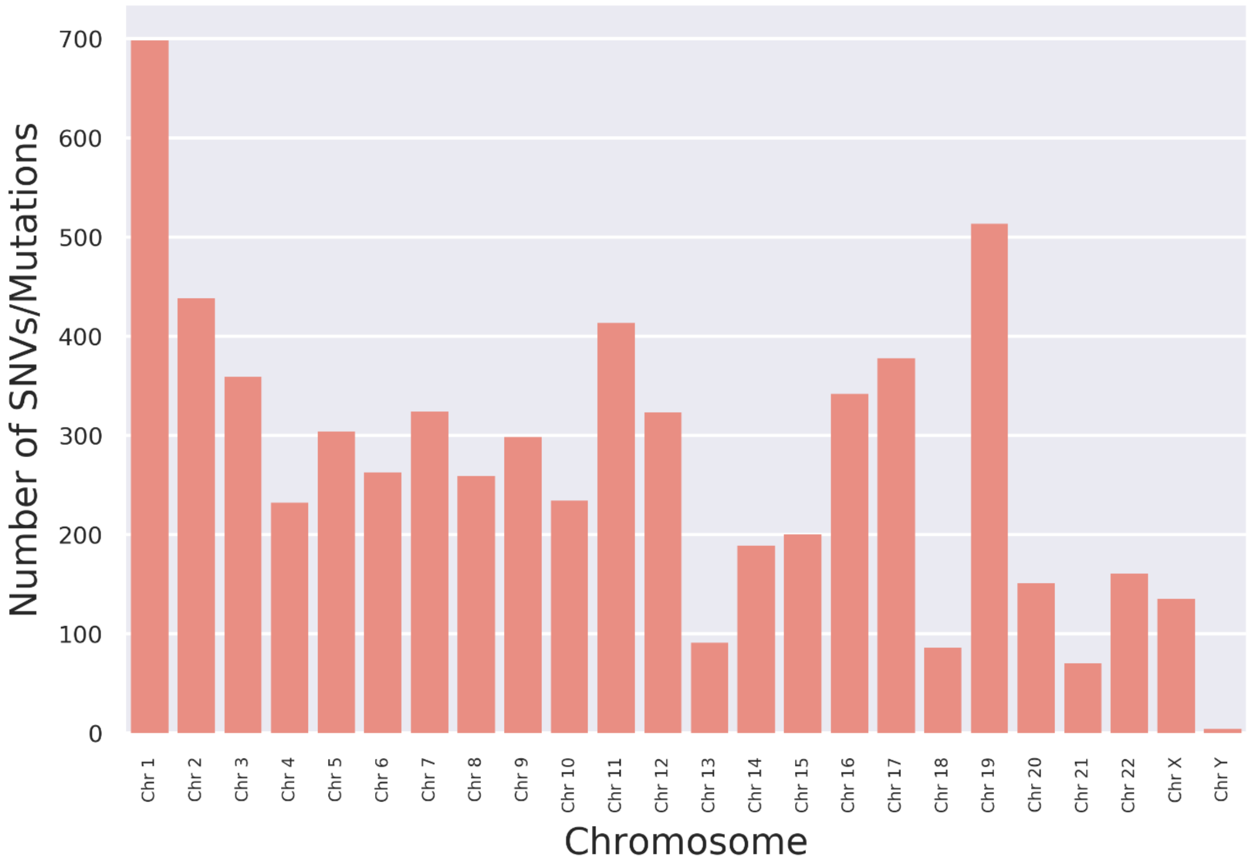

Supplementary Figure S3. The numbers of mutations and SNVs in each chromosome are illustrated in the whole-exome sequencing dataset of effusion-associated DLBCL. They are different in various chromosomes.

Supplementary Figure S4. Genes with higher frequency (>3) of mutations/SNVs

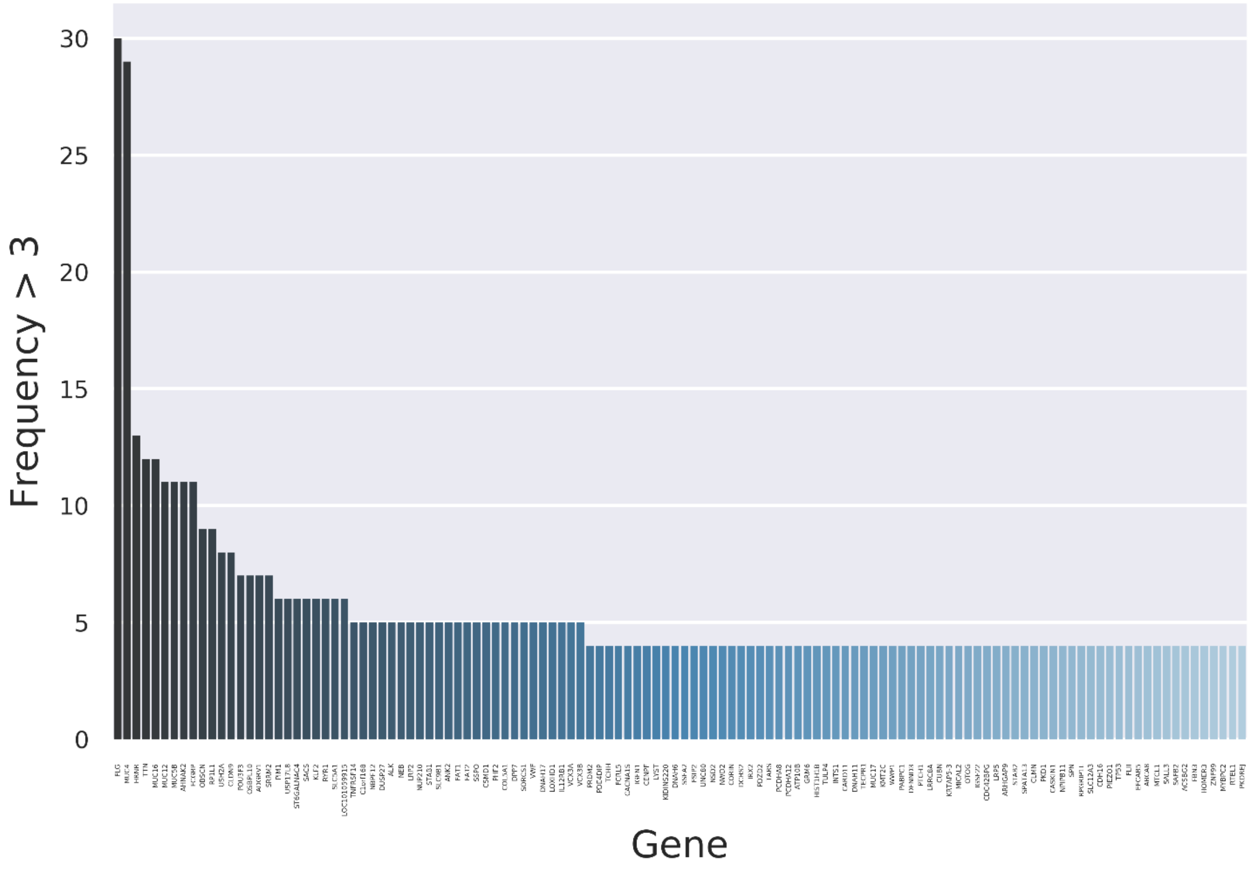

Supplementary Figure S4. The genes that their number of mutations/SNVs is higher than three in the effusion-associated DLBCL dataset. These genes are more likely to be the genes associated with tumors in effusions than the genes with lower frequency in the number of mutations/SNVs.

Supplementary Figure S5. Genes with higher accumulation scores (>1)

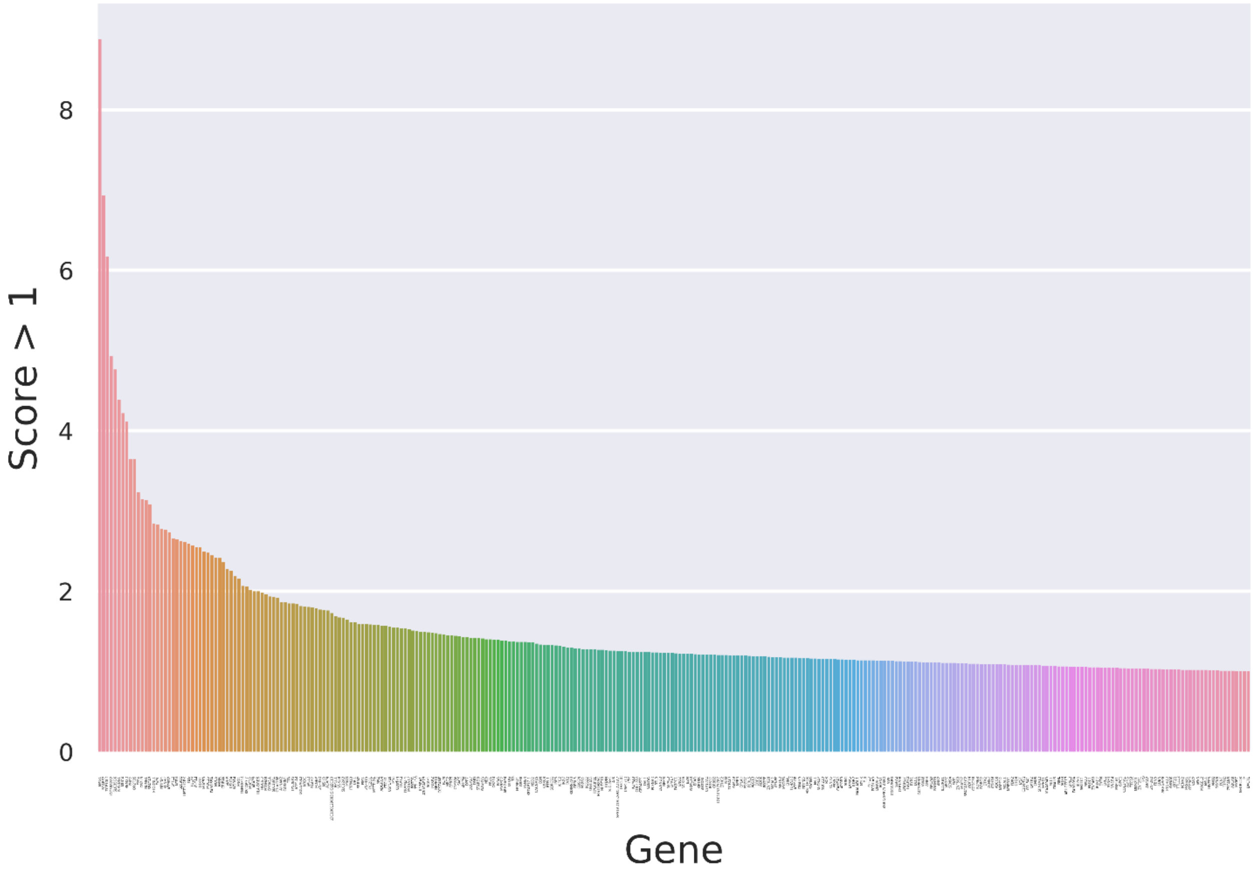

Supplementary Figure S5. As explained in the text, we proposed a mutational accumulation-based method to extract tumor-in-effusions-associated genes. Hence, we calculated the accumulation score of each gene in the effusion-associated DLBCL dataset. The genes with higher accumulation scores were considered as tumor-in-effusions-associated candidates. This figure shows the genes that have an accumulation score higher than one, which consist of many glycoproteins and transmembrane proteins.

Supplementary Figure S6. Relative frequency of base substitutions in both effusion-associated DLBCL and non-effusion-associated DLBCL cohorts

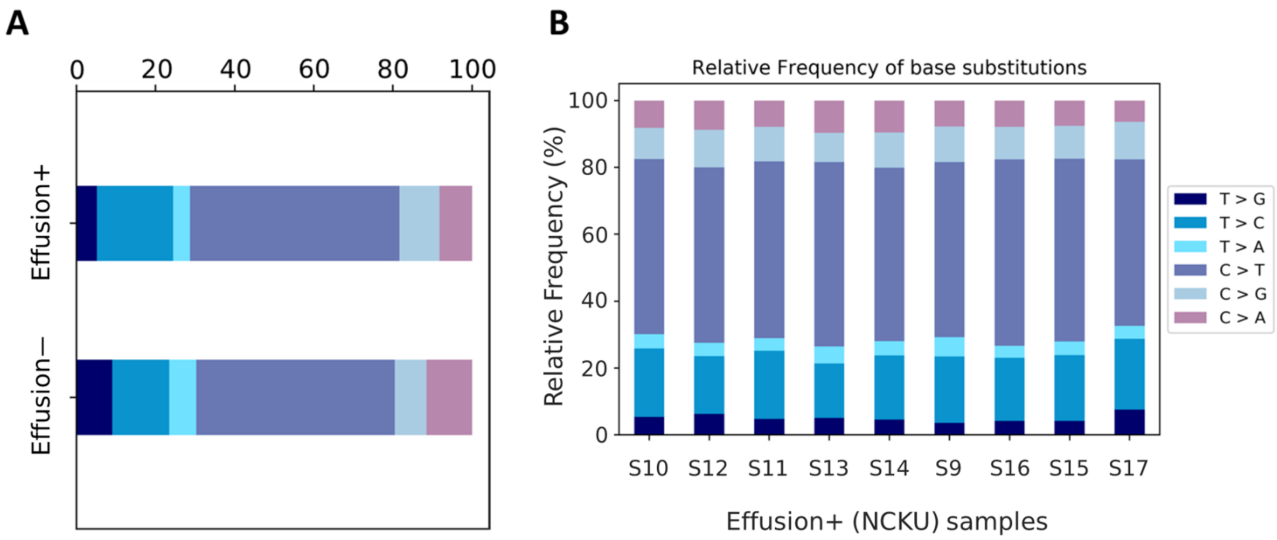

Supplementary Figure S6. Base excision repair (BER) and nucleotide excision repair (NER) are categories of DNA repair that could be recognized by mutational pattern. Defects in BER and NER components result in higher rates of C-to-T and T-to-C [8-9]. (A) The relative frequency of base substitutions in both effusion-associated (effusion+) DLBCL and non-effusion-associated (effusion—) DLBCL cohorts reveals that samples with tumors in effusions have higher rates in T-to-C, C-to-T, and C-to-G compared to the samples with tumors in solid organs. (B) Relative frequency of base substitutions in different samples of the effusion-associated DLBCL dataset (tumors in effusions).

Supplementary Figure S7. Stacked bar charts of SNVs and indels in the effusion-associated DLBCL and non-effusion-associated DLBCL cohorts

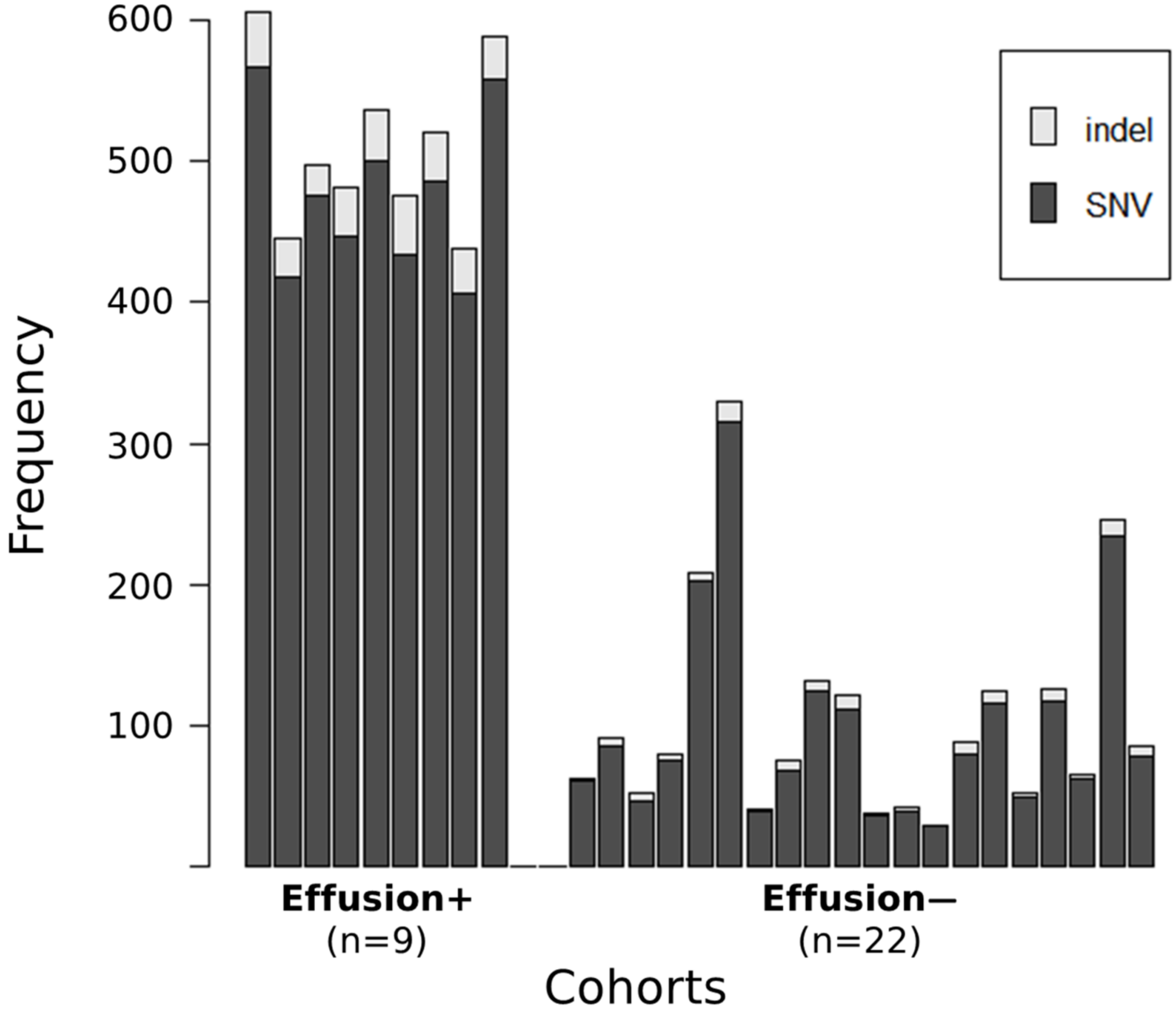

Supplementary Figure S7. Comparison of insertion-deletion (Indel) and SNV counts in the effusion-associated (effusion+) DLBCL and non-effusion-associated (effusion—) DLBCL datasets.

Supplementary Figure S8. All genes affected by mutations in at least one effusion-associated DLBCL sample

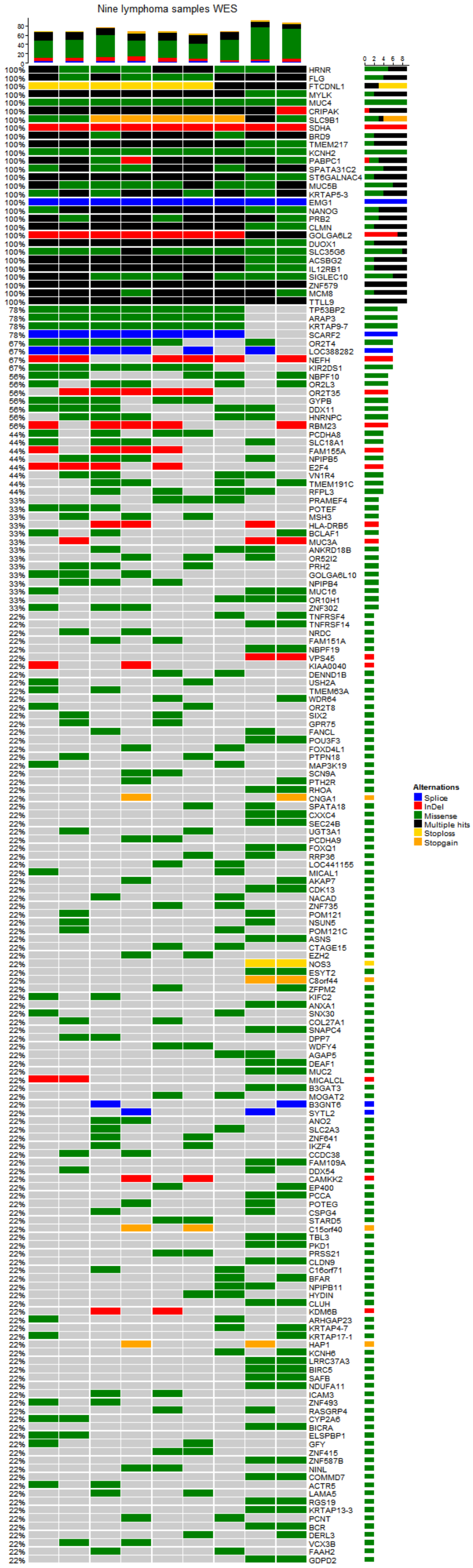

Supplementary Figure S8. Genes affected by splice, indels, missense, multiple hits, stop-loss, and stop-gain mutations in at least one effusion-associated DLBCL sample.

Supplementary Figure S9. Pathways significantly activated in samples bearing tumor cells in effusions by GSEA

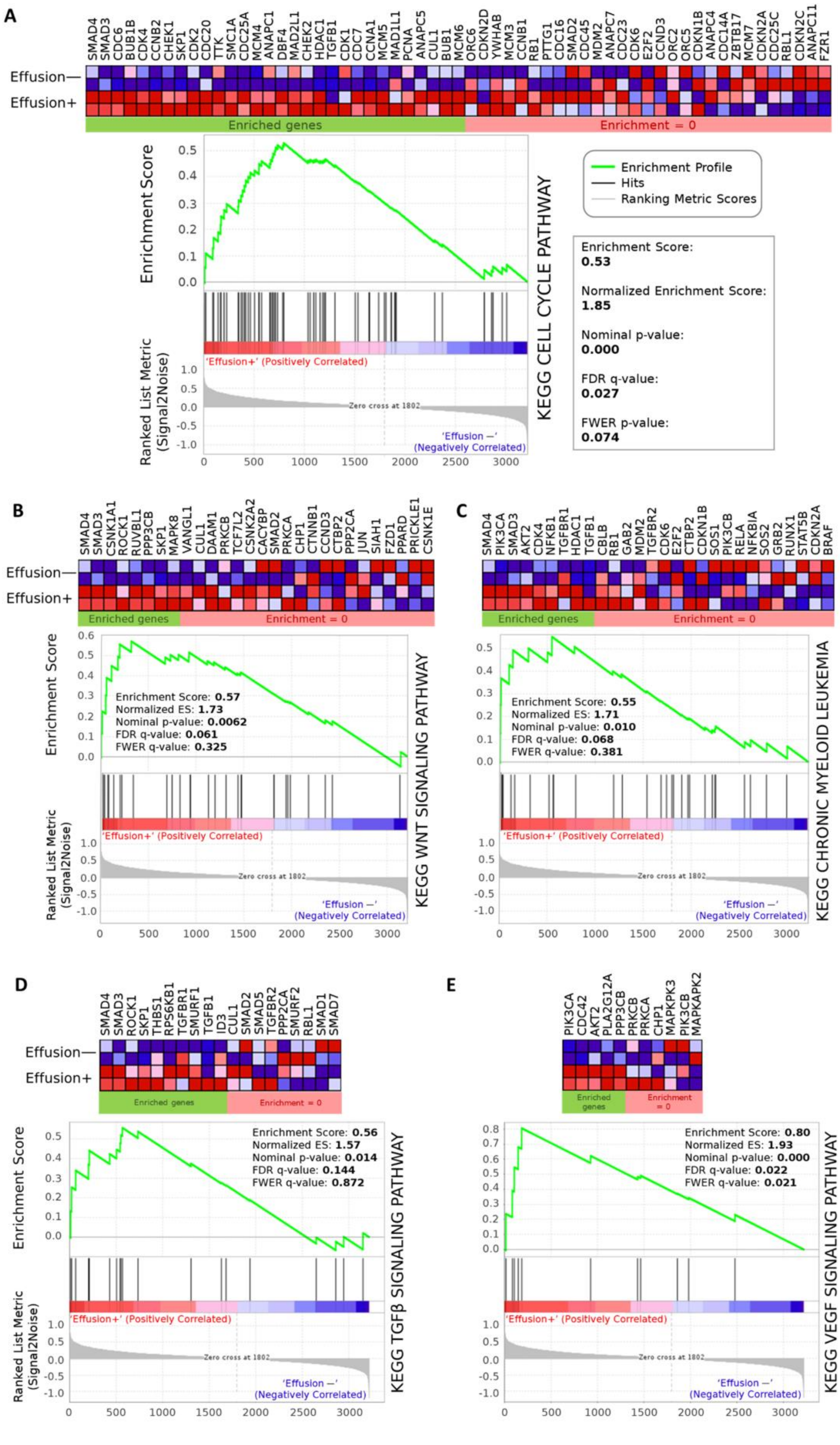

Supplementary Figure S9. Gene set enrichment analysis (GSEA) on KEGG gene set reveals that samples bearing tumors in effusions are positively associated with the (A) Cell Cycle pathway, (B) WNT signaling, (C) chronic myeloid leukemia, (D) TGFβ, (E) VEGF pathways.

Supplementary Figure S10. SUMO pathway by IPA

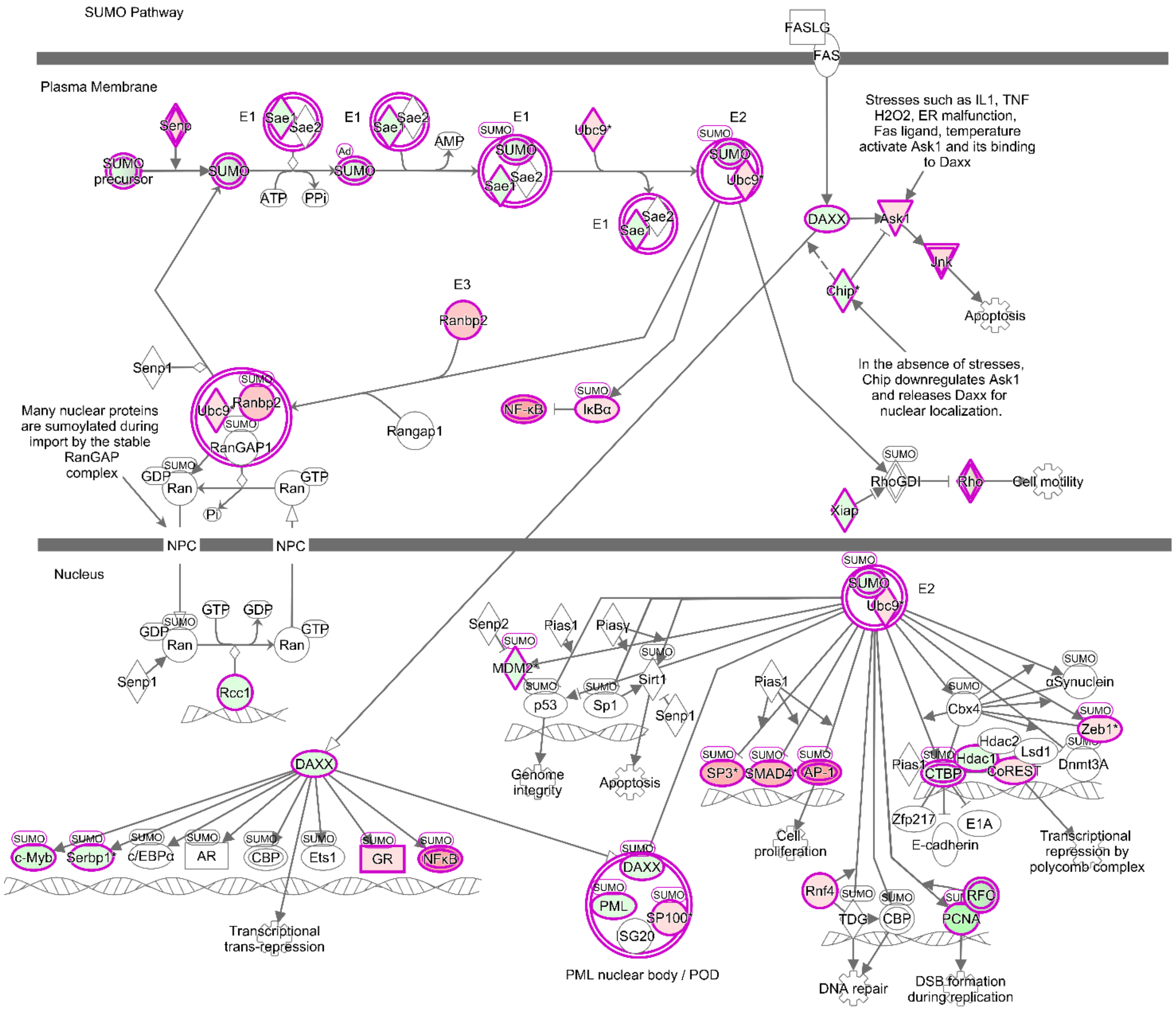

Supplementary Figure S10. IPA findings reveal that samples with tumors in effusions are positively associated with the activation of SUMOylation pathway.

Supplementary Figure S11. p38 MAPK-associated signaling pathways by IPA

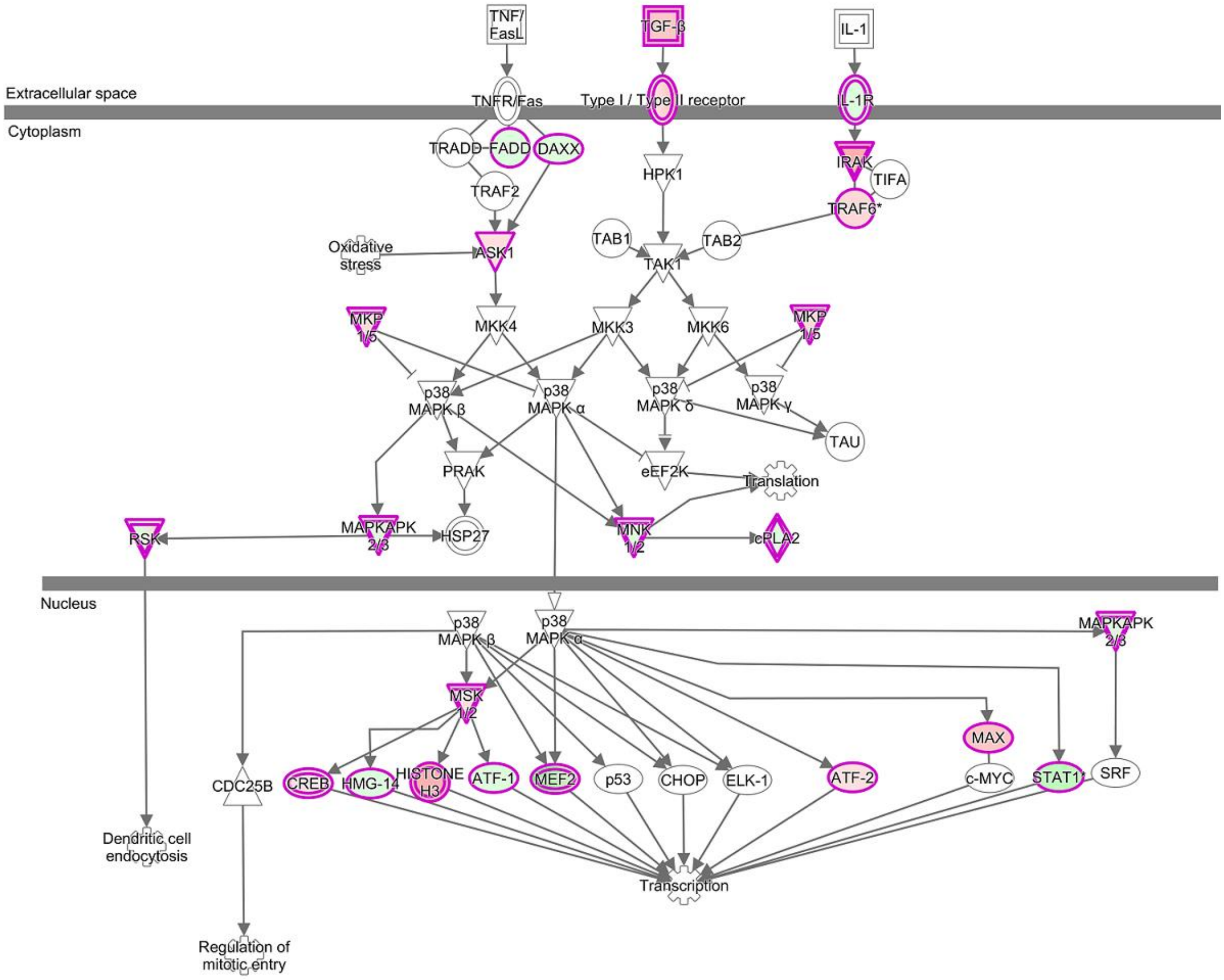

Supplementary Figure S11. IPA findings reveal that samples with tumors in effusions are positively associated with the activation of p38 MAPK signaling.

Supplementary Figure S12. DNA replication pathway by IPA

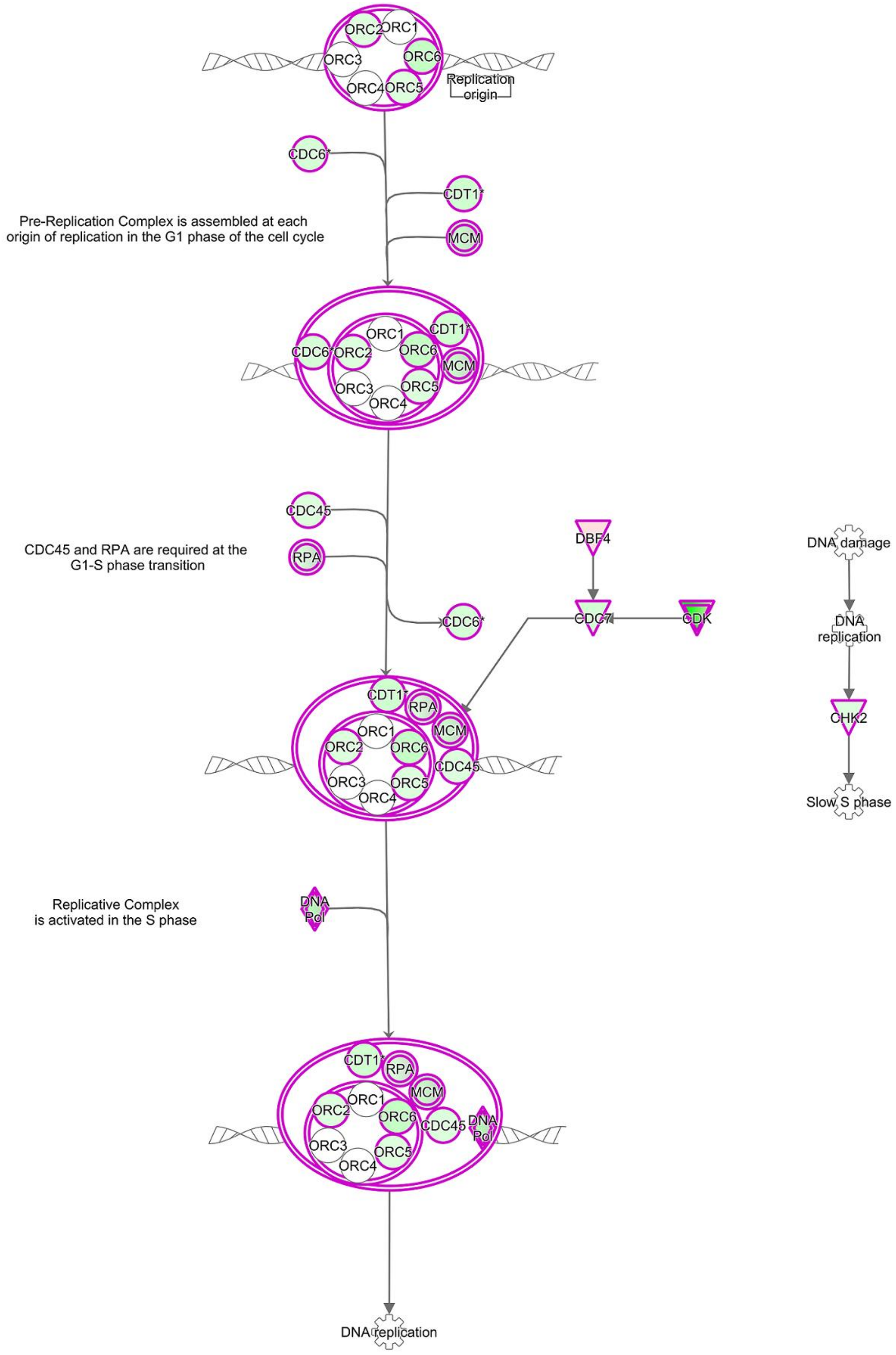

Supplementary Figure S12. IPA findings reveal that samples with tumors in effusions are positively associated with the inhibition of the cell cycle control of chromosomal replication pathway.

Supplementary Figure S13. Nucleotide excision repair (NER) signaling pathway by IPA

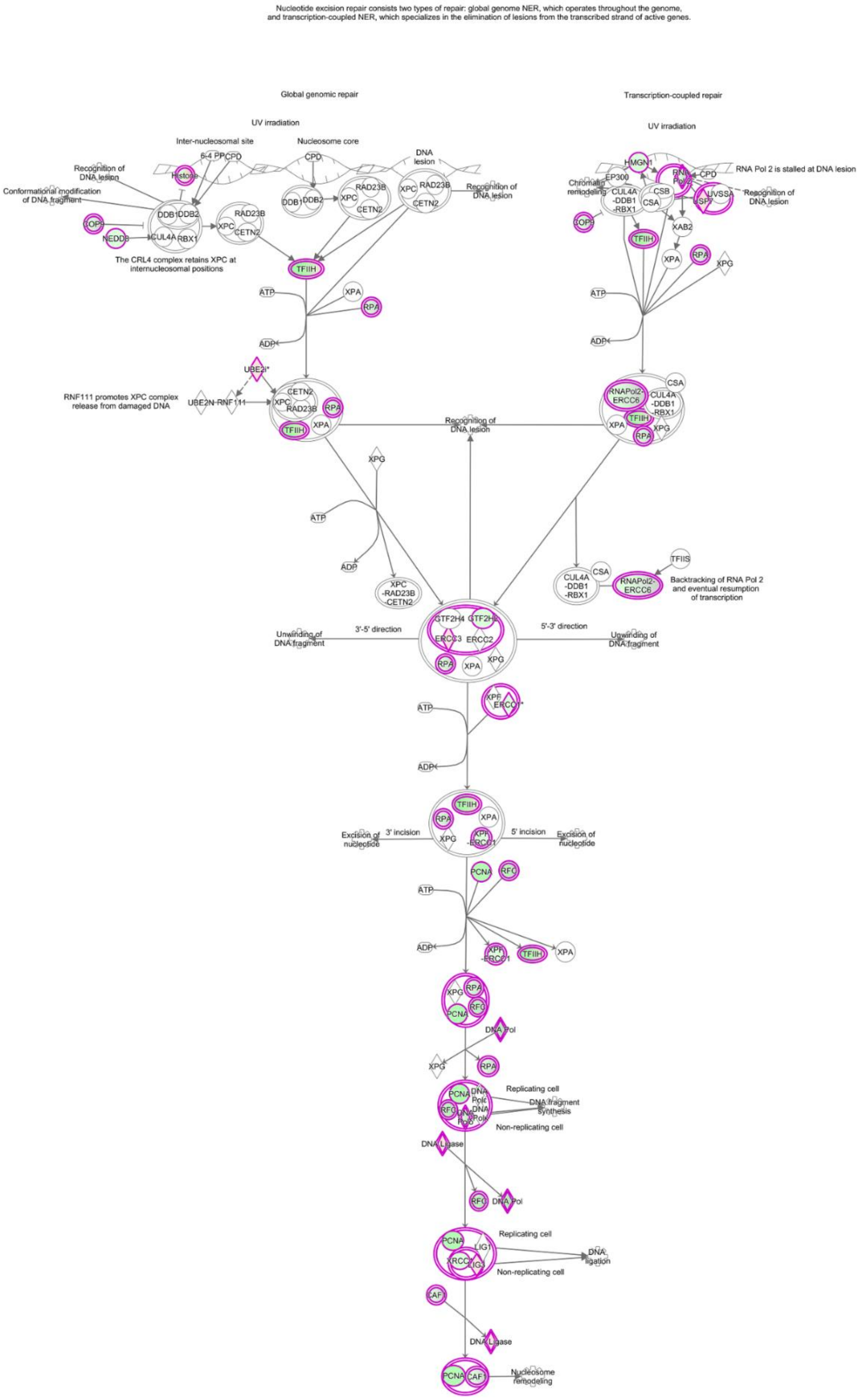

Supplementary Figure S13. IPA findings reveal that samples with tumors in effusions are positively associated with the inhibition of nucleotide excision repair (NER) pathway.

Supplementary Figure S14. Cell cycle checkpoint control pathway by IPA

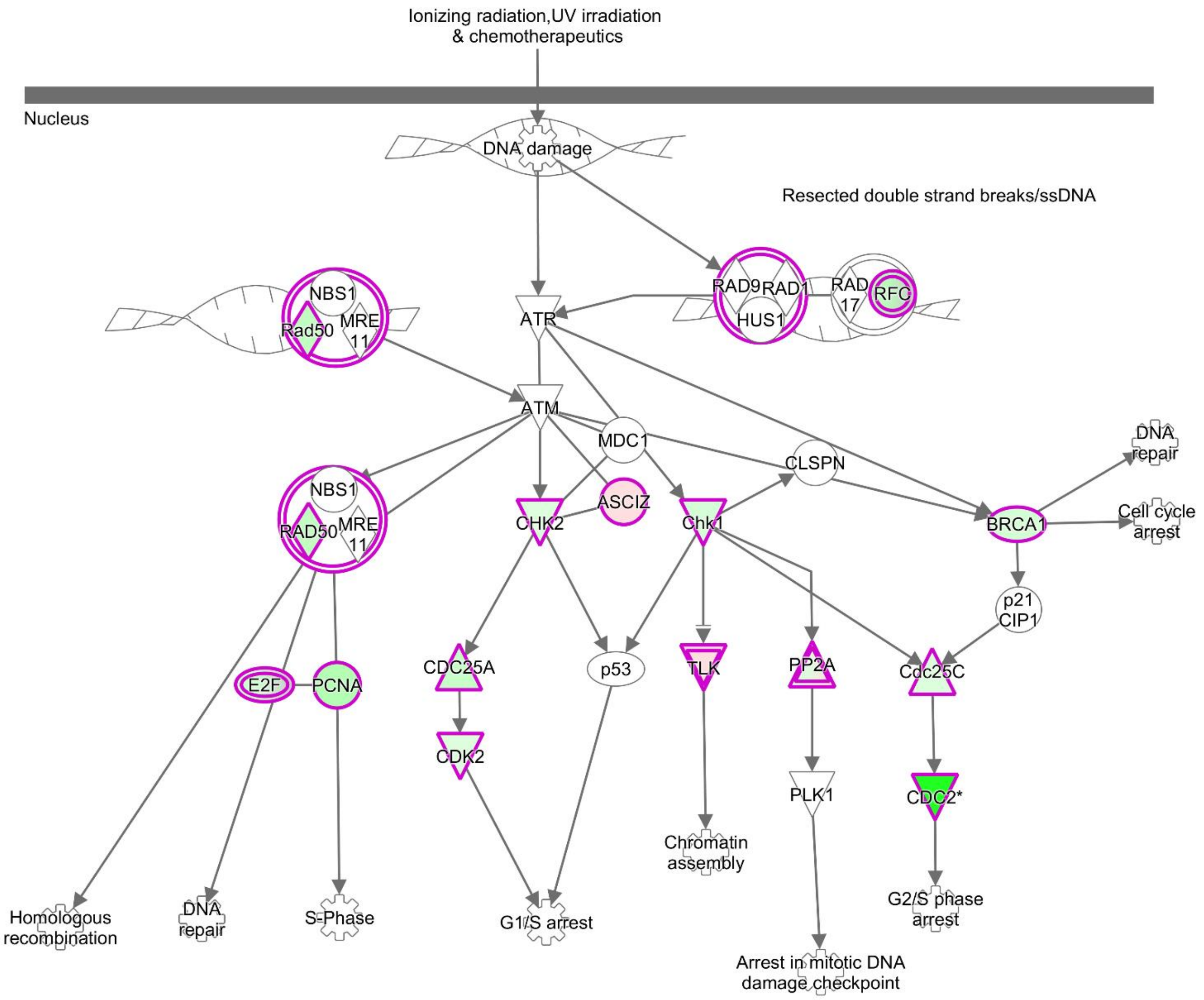

Supplementary Figure S14. IPA findings reveal that samples with tumors in effusions are positively associated with the inhibition of the checkpoint kinase (CHK) proteins in cell cycle checkpoint control.

Supplementary Figure S15. p53-related signaling pathways by IPA

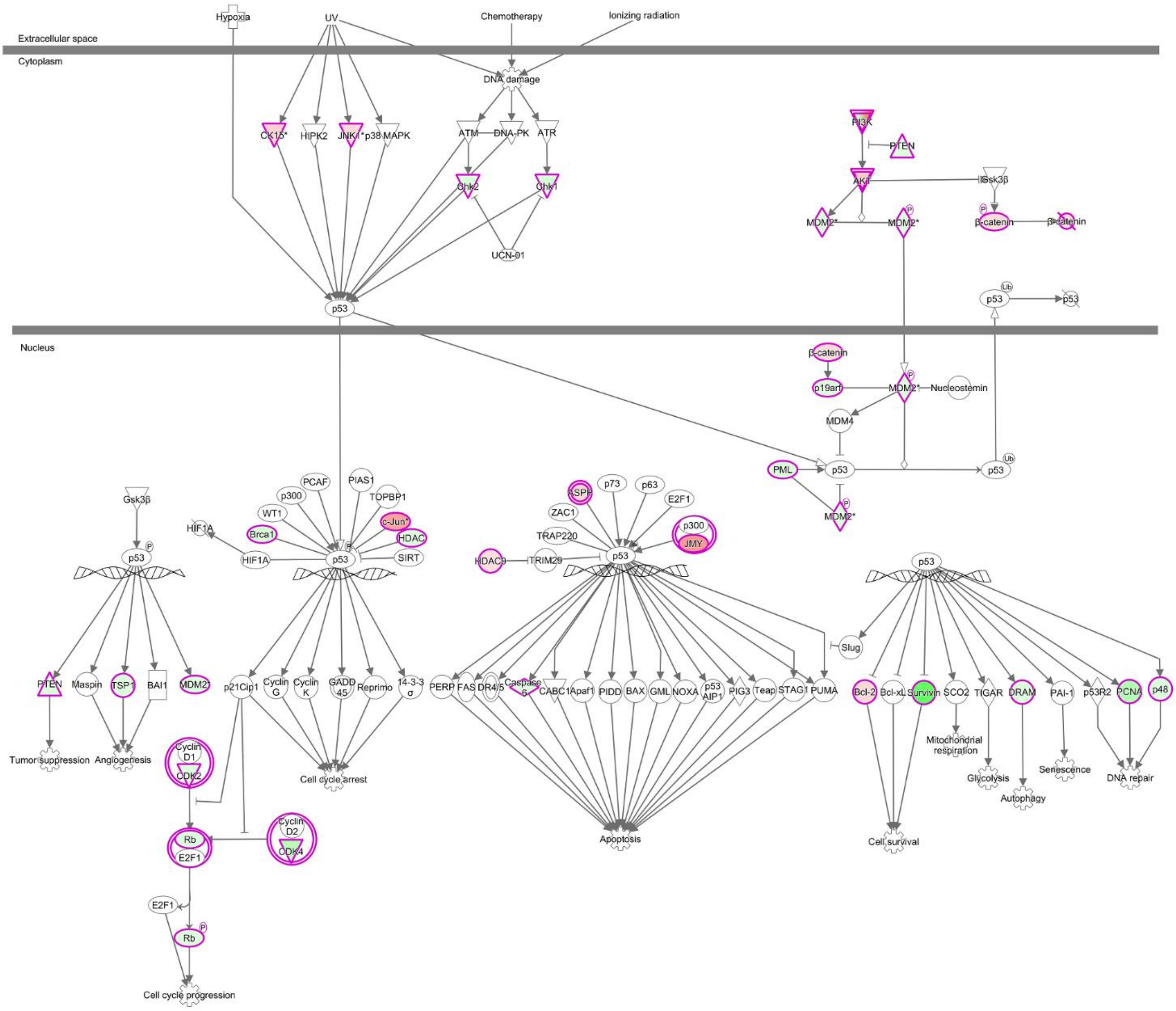

Supplementary Figure S15. IPA findings reveal that samples with tumors in effusions are positively associated with the inhibition of p53 pathway. Solid lines signify direct interactions, dotted lines depict indirect interactions. Arrows from one node to another indicate that the node acts upon the other. Lines without arrows represent binding. Node shapes are vertical diamond indicates enzyme; dotted rectangle indicates ion channel; inverted triangle indicates kinase; horizontal diamond indicates peptidase; triangle indicates phosphatase; horizontal oval indicates transcription regulator; double-circle indicates complex/group; trapezium indicates microRNA; semicircle indicates mature microRNA; the circle indicates other.

Supplementary Figure S16. Expression of HDAC1 and MDM2 in DLBCL without lymphomatous effusions and clinicopathologic factors affecting overall survival of patients with DLBCL

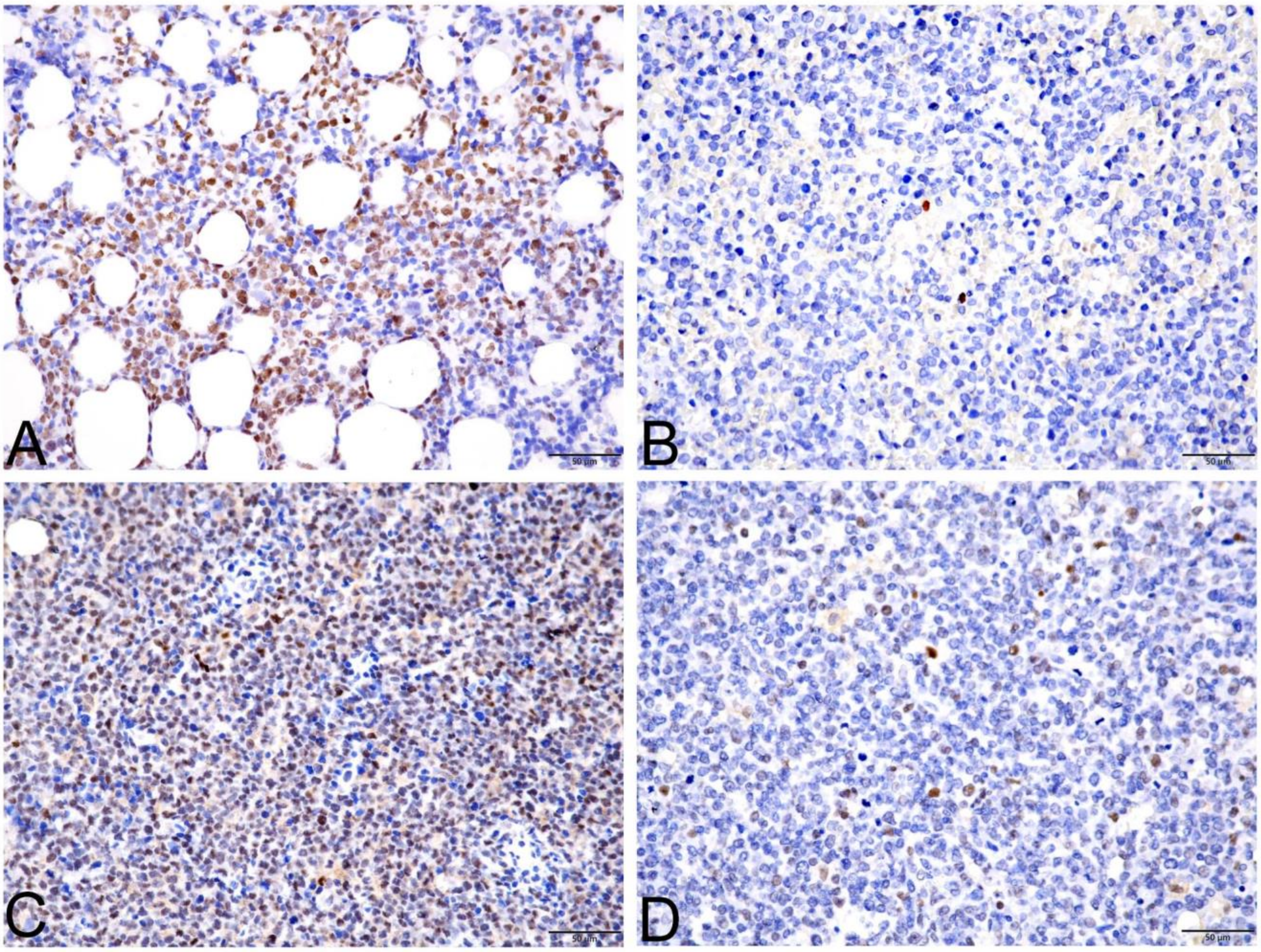

Clinicopathologic parameters affecting overall survival

Parameter	Worse factor	No. (%)	p value
Sex	Male	27 (53%)	0.508
Age	>60 years	28 (55%)	0.005
LDH	>200 IU/L	39 (80%)	0.140
IPI score	3~5	28 (55%)	<0.001
Stage	III-IV	33 (66%)	0.110
MDM2	Present	18 (35%)	0.571
HDAC1	Present	27 (53%)	0.005
Effusions	Present	31 (61%)	<0.001

Supplementary Figure S16. Representative IHC staining of HDAC1 (A, positive; B, negative) and MDM2 (C, positive; D, negative) in an independent cohort of DLBCL cases without lymphomatous effusions, respectively. Scale bars denote 50 µm. The presence of lymphomatous effusions is a more powerful factor for predicting poor survival than HDAC1 expression.
